# G6PD deficiency and the exportome of *plasmodium falciparum* FCB-2: A comparative analysis utilizing one-dimensional blue-native electrophoresis and timstof mass spectrometry

**DOI:** 10.1007/s00436-026-08717-y

**Published:** 2026-07-02

**Authors:** Isaac De la Rosa Cogollo, María Luisa Hernáez, Luis Felipe Clemente, Albeiro Marrugo-Padilla, Erika Rodríguez-Cavallo, Darío Méndez-Cuadro

**Affiliations:** 1 Faculty of Exact and Natural Sciences, Campus of San Pablo. Analytical Chemistry and Biomedicine Group, University of Cartagena, Cartagena de Indias, Colombia; 2 Proteomics Unit, Faculty of Pharmacy, Complutense University of Madrid (UCM), Madrid, Spain; 3 Faculty of Pharmaceutical Sciences, Analytical Chemistry and Biomedicine Group, Cartagena de Indias, University of Cartagena, Cartagena de Indias, Colombia

**Keywords:** *Plasmodium falciparum* exportome, G6PD deficiency, Blue-Native, LC-MS-tims-TOF

## Abstract

*Plasmodium falciparum*, the etiological agent of severe malaria in humans, remodels the surface of infected erythrocytes to evade host immune defenses and ensure its survival. During its intraerythrocytic development, *P. falciparum* modifies the composition of protein complexes within the host cell membrane and cytoskeleton by exporting parasitic proteins to the surface of the infected erythrocyte. However, there is limited understanding of its behavior in cells harboring protective polymorphisms against severe malaria, such as those associated with glucose-6-phosphate dehydrogenase (G6PD) deficiency, where reduced adhesion and early phagocytosis have been observed due to oxidative damage to the surface proteins of parasitized erythrocytes. Nonetheless, the impact of G6PD deficiency on the exportome proteins of *P. falciparum* in infected erythrocytes remains to be elucidated. In this in vitro study, we conducted a comparative analysis of the *P. falciparum* FCB-2 exportome in G6PD-deficient versus normal human red blood cells. Two membrane protein conglomerates were isolated at the ring, trophozoite, and schizont stages using Blue-Native electrophoresis, followed by in-gel digestion and LC-MS-tims-TOF analysis. The analysis revealed a significant decrease in the number of *P. falciparum* proteins identified in all asexual stages for both protein conglomerates on the surface of infected G6PD-deficient erythrocytes. A decrease in the number of effector, adhesion, trafficking, and signaling proteins that reinforce the protective mechanisms previously described was observed. These findings provide new insights into the pathways for understanding the molecular events that support G6PD’s protective mechanisms against malaria.

**Supplementary Information:**

The online version contains supplementary material available at https://doi.org/10.1007/s00436-026-08717-y.

## Introduction

Malaria, or paludism, is a life-threatening disease caused by protozoa belonging to the genus *Plasmodium*. Among these species, *Plasmodium falciparum* and its intraerythrocytic asexual cycle are the primary contributors to morbidity and mortality in humans (Julien and Wardemann 2019). During its asexual development, *P. falciparum* reorganizes the membrane and cytoskeletal complexes of the infected erythrocyte, coincident with the parasite’s progression through its intraerythrocytic stages. These modifications result from the export of parasitic proteins to the host’s membrane and cytosol. The exported proteins enhance the cell’s permeability and rigidity while facilitating the installation of parasite adhesins, such as *P. falciparum* erythrocyte membrane protein 1 (PfEMP1), on the surface of red blood cells (RBCs) (McHugh et al. 2020). This mechanism promotes parasite survival within the microvasculature by enabling evasion of splenic clearance contributing to the organ-specific pathology associated with severe malaria. The exported proteins are crucial for remodeling the erythrocyte surface and for evading the host immune response (Schwarzer and Skorokhod 2024).

The transport of proteins from the parasite to the erythrocyte membrane is mediated by a complex export system facilitated by the Protein Translocation Export Complex (PTEX), a multi-subunit protein channel integrated into the parasitophorous vacuole membrane (PVM). The core of this translocon comprises an ATP-driven unfoldase (HSP101), a membrane-spanning pore (EXP2), and a scaffolding protein (PTEX150), along with the auxiliary components PTEX88 and TRX2 (de Koning-Ward et al. 2009). PTEX is essential for the translocation of proteins from the parasitophorous vacuole into the erythrocyte cytoplasm, thereby ensuring their proper localization and functionality (Kilili and LaCount 2011). Consequently, disruption of this export system may impair the parasite’s capacity to remodel the erythrocyte, leading to a reduction in virulence.

Among the most extensively studied exported proteins is *Pf*EMP1 (*Plasmodium falciparum* erythrocyte membrane protein 1), which anchors to the surface of the infected erythrocyte and facilitates its adhesion to endothelial receptors such as ICAM-1 and CD36. This protein exhibits high variability, allowing the parasite to evade the adaptive immune response (McHugh et al. 2020; Wahlgren et al. 2017). Additionally, proteins such as RESA (Ring-Infected Erythrocyte Surface Antigen) play a critical role in stabilizing the erythrocyte cytoskeleton and protecting it from mechanical stress during circulation (Mills et al. 2007). Other protein groups, including heat shock proteins (HSPs), are also involved in the transport and proper folding of these exported molecules (Beck and Ho 2021).

This parasitic protozoan has exerted selective pressure in malaria-endemic regions, resulting in the emergence of erythrocytic polymorphisms, such as glucose-6-phosphate dehydrogenase deficiency (d-G6PD). This polymorphism is linked to an adaptive response to parasitic infection, providing a degree of protection against the severe clinical manifestations of malaria (Liang et al. 2020; Shin et al. 2024). Given that G6PD-deficient erythrocytes infected with *P. falciparum* exhibit evidence of oxidative modifications of host membrane proteins (Contreras-Puentes et al. 2019; Méndez et al. 2011), this altered redox environment likely destabilizes the host-parasite protein interactions required for surface remodeling. These alterations have the potential to attenuate cytoadhesion and virulence in G6PD deficient cells. Nonetheless, the underlying mechanisms of this tolerance remain to be fully elucidated. This highlights the need to investigate how the composition of the erythrocyte membrane and its underlying cytoskeleton is altered by *Plasmodium falciparum* exported proteins throughout the stages of its asexual life cycle in G6PD deficient cells.

In order to contribute to elucidating these mechanisms, we conducted a comparative study using synchronous *P. falciparum* cultures at high parasitemia, combined with Blue-Native Page (BN-PAGE), liquid chromatography, and timsTOF mass spectrometry.

## Materials and methods

### Isolation of the erythrocyte pack

Peripheral venous blood samples were collected in EDTA tubes from healthy male subjects. This group included both G6PD A− hemizygous individuals and enzymatically normal controls. Specifically, the study involved two enzymatically healthy volunteers and two individuals diagnosed with G6PD deficiency (d-G6PD), all of whom resided in Cartagena, Colombia. Written informed consent was obtained from all participants in accordance with the ethical guidelines established in the Declaration of Helsinki and Resolution 8430 of 1993 by the Ministry of Health of the Republic of Colombia.

The fresh samples were centrifugated at 2300 rpm for 10 min at 20 °C to remove the plasma fraction. The leukocyte layer was subsequently removed using a Lymphoprep (Axis-Shield) density gradient. The resulting erythrocyte pack was resuspended in complete RPMI-1640 medium at a 1:1 ratio for the in vitro maintenance of *Plasmodium falciparum* FCB-2 strain cultures (Radfar et al. 2009).

### Synchronous cultures of *plasmodium falciparum* FCB-2 and identification of parasite stages

**Fig. 1:**
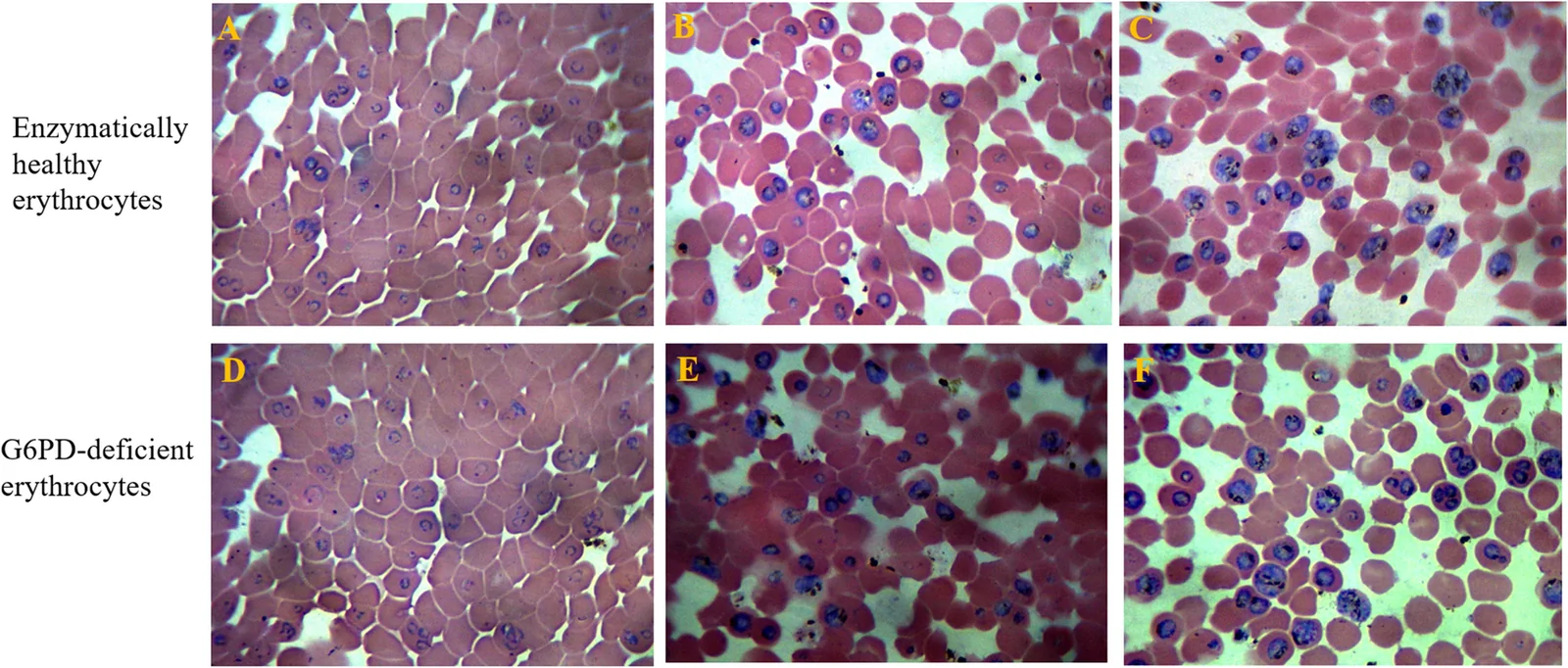
Synchronized cultures of *Plasmodium falciparum FCB-2* were established in both enzymatically healthy and G6PD-deficient erythrocytes. Wright-stained thin blood smears reveal the presence of ring forms at 10–14 hpi (A, D), trophozoites at 24–28 hpi (B, E), and schizonts at 38–42 hpi (C, F) within enzymatically healthy erythrocytes (A - C) and G6PD-deficient erythrocytes (D - F). Cultures exhibiting a predominant stage-specific morphology were selected for subsequent proteomic analyses

Synchronous cultures of *Plasmodium falciparum FCB-2* with high parasitemia (approximately 25% – 30%) were established in both enzymatically healthy and G6PD-deficient erythrocytes, following the protocol outlined by Radfar et al. (2009). The developmental stages of the parasite were monitored through microscopic examination of thin blood smears stained with Wright’s stain. Ring forms were identified by small intraerythrocytic annular structures, observable approximately 10–14 h post-invasion (hpi). Trophozoites were characterized by an increase size, as evidenced by visible pigment accumulation and enhanced cytoplasmic volume, observed between 24 and 28 hpi. Schizonts were identified by a further increase in cytoplasmic volume appearing between 38 and 42 hpi (see Fig. 1). Only the infected cell cultures exhibiting a predominant morphology corresponding to the targeted developmental stage were preserved at -80 °C for subsequent proteomic assays.

### Extraction of membrane complexes from infected erythrocytes

Erythrocyte fractions infected with *Plasmodium falciparum* (ring, trophozoite, and schizont stages) were treated with 5% sorbitol in 20 mM sodium HEPES buffer (pH 7.4). This was followed by vigorous vortexing for approximately 45 s and incubation at 37 °C for 10 min to facilitate the lysis of the infected erythrocytes. The samples were then centrifuged at 1700 rpm for 6 min at 4 °C, the resulting supernatant, containing both infected erythrocyte membrane proteins and soluble proteins, was collected. The pellet underwent two additional wash cycles with sorbitol to ensure comprehensive protein extraction (Méndez et al. 2011).

The sorbitol lysates, enriched with erythrocyte membranes and *Plasmodium* proteins, were subjected to five washing cycles (10.000 rpm, 4 °C, 20 min) with cold 5 mM phosphate buffer to isolate the erythrocyte ghosts, following a previously established protocol (Méndez et al. 2011). To preserve their native conformations, ghosts were dissolved in a native sample buffer (see below) and stored at -80 °C until utilized in proteomic assays involving Blue-Native electrophoresis.

### Sample preparation for blue-native electrophoresis (BN-PAGE)

For BN-PAGE, protein complexes were solubilized in a buffer containing 50 mM Bis-Tris (pH 7.0), 750 mM ε-aminocaproic acid, and 0.5 mM Na_2_EDTA, supplemented with 20% dodecyl-β-D-maltoside (DDM). Depending on the extracted volume, one-fifth volume of 50% glycerol and one-twelfth volume of 5% Coomassie G250 were incorporated into the sample prior to loading onto the gel (Aref et al. 2025; van Gestel et al. 2010; Wittig et al. 2006). The Blue-Native gels were prepared with fixed percentages: a 3% stacking gel and an 8% resolving gel. Electrophoretic separation was performed at a constant voltage of 250 V for approximately 10 h at 4 °C, with temperature control provided by a thermostatic cooling bath system (LAUDA Alpha RA12).

### Protein identification by LC-MS

#### Extraction of tryptic peptides

Bands of protein conglomerates 1 and 2 from each lane of the one-dimensional blue native (1D BN) gels were excised and subjected to in-gel digestion using the iST kit (PreOmics GmbH, Martinsried, Germany) with minor modifications. Briefly, gel slices were dehydrated through acetonitrile washes and dried under vacuum in a SpeedVac for 15 min. The gel slices were then incubated at 56 °C for 30 min in a solution of 10 mM dithiothreitol (DTT) as a reducing agent.

Next, DTT was removed and the gel slices were washed with acetonitrile to eliminate any residual reducing agent. The slices were then incubated with 55 mM iodoacetamide for 15 min in the dark. Following this incubation, the iodoacetamide was removed, and the slices were incubated for 10 min with acetonitrile, followed by an additional 10-minute incubation in 50 mM ammonium bicarbonate (AMBIC). Acetonitrile was then added to the AMBIC without removal, and the resulting mixture was incubated for 15 min. After all liquids were removed, the gel slices were dried in a SpeedVac for 45 min.

Peptide digestion was performed using trypsin at a ratio of 1 µg trypsin per 20 µg protein. The gel pieces were incubated at 4 °C for 45 min. Afterward, the trypsin solution was removed, and the gels were incubated with AMBIC at 37 °C overnight.

The following day, the tubes were equilibrated at room temperature for 15 min and then centrifuged at 13,000 rpm. The supernatant was transferred into new centrifuge tubes. To enhance peptide extraction, the gel pieces underwent additional washes with acetonitrile, AMBIC, and acetonitrile for 5 min each. The resulting supernatants were pooled with the initial fraction. Finally, the samples were dried for 2 h in a SpeedVac and reconstituted in 100 µL for subsequent analyses.

#### Mass spectrometry and LC-MS/MS data acquisition

Samples were analyzed using a high-resolution timsTOF Pro 2 mass spectrometer (Bruker Daltonics) combined with an EvoSep One liquid chromatography platform (EvoSep Biosystems). For each sample, 200 ng of peptides were loaded onto Evotip Pures (EvoSep Biosystems). Chromatography was conducted using the 15 SPD method, characterized by a gradient length of 88 min and a flow rate of 0.22 µL/min. The mass spectrometer operated in Data-Dependent Acquisition with Parallel Accumulation and Serial Fragmentation (DDA-PASEF) mode, with a cycle time of 1.17 s and a trapped ion mobility ramp time of 100 ms. The mass-to-charge (m/z) scan range was set between 100 and 1700 m/z.

#### Data processing

Raw files (*.d) obtained from the mass spectrometry analysis utilizing DDA-PASEF were processed through the FragPipe v20 interface, integrated with the MSFragger proteomics search engine. Database searches were conducted against a concatenated UniProt database comprising the proteomes of both *Homo sapiens* and *Plasmodium falciparum 3D7*, which was downloaded in September 2023. Protein identification was based on peptide-spectrum matches, and the origin of proteins was assigned according to the taxonomic annotations provided by UniProt (UniProt 2025), facilitating the differentiation between host erythrocyte and parasite-derived proteins. The search parameters included: trypsin digestion with a tolerance for up to two missed cleavages, a minimum peptide length of eight amino acids, a precursor mass tolerance ± 20 ppm, and an isotope error set to 0/1/2. Carbamidomethylation of cysteine (+ 57.021465 Da) was designated as a fixed modification. For subsequent exportome analysis, only proteins taxonomically assigned to *Plasmodium falciparum* were retained.

The mass spectrometry data have been deposited in the ProteomeXchange Consortium via the PRIDE repository under the identifier PXD061301.

#### Functional classification analysis

Identified *Plasmodium falciparum* proteins were classified according to their primary biological functions and processes, using Gene Ontology terms annotated in the UniProtKB database (UniProt 2025). This classification was further refined using PlasmoDB tool, a component of the VEuPathDB bioinformatics platform dedicated to eukaryotic pathogens, vectors, and hosts. (Alvarez-Jarreta et al. 2024).

## Results

The primary objective of this study was to identify *Plasmodium falciparum* proteins anchored or embedded in the membranes of G6PD-deficient erythrocytes across the asexual stages: rings, trophozoites, and schizonts. It is well established that the expression of *Plasmodium* proteins during its intraerythrocytic asexual cycle varies with the developmental phase (Beck and Ho 2021).

To identify *Plasmodium* proteins, we prepared native membrane ghosts from both G6PD-deficient erythrocytes and control samples infected in synchronous high-parasitemia cultures at all three stages. To validate the quality of these native erythrocyte membrane ghosts, a fraction was subjected to 1% SDS and SDS-PAGE electrophoresis. The resulting electrophoretic profiles (Figure S1_SDS-PAGE) were consistent across all samples, confirming the integrity of the infected erythrocyte membrane ghosts used in this study.

**Fig. 2:**
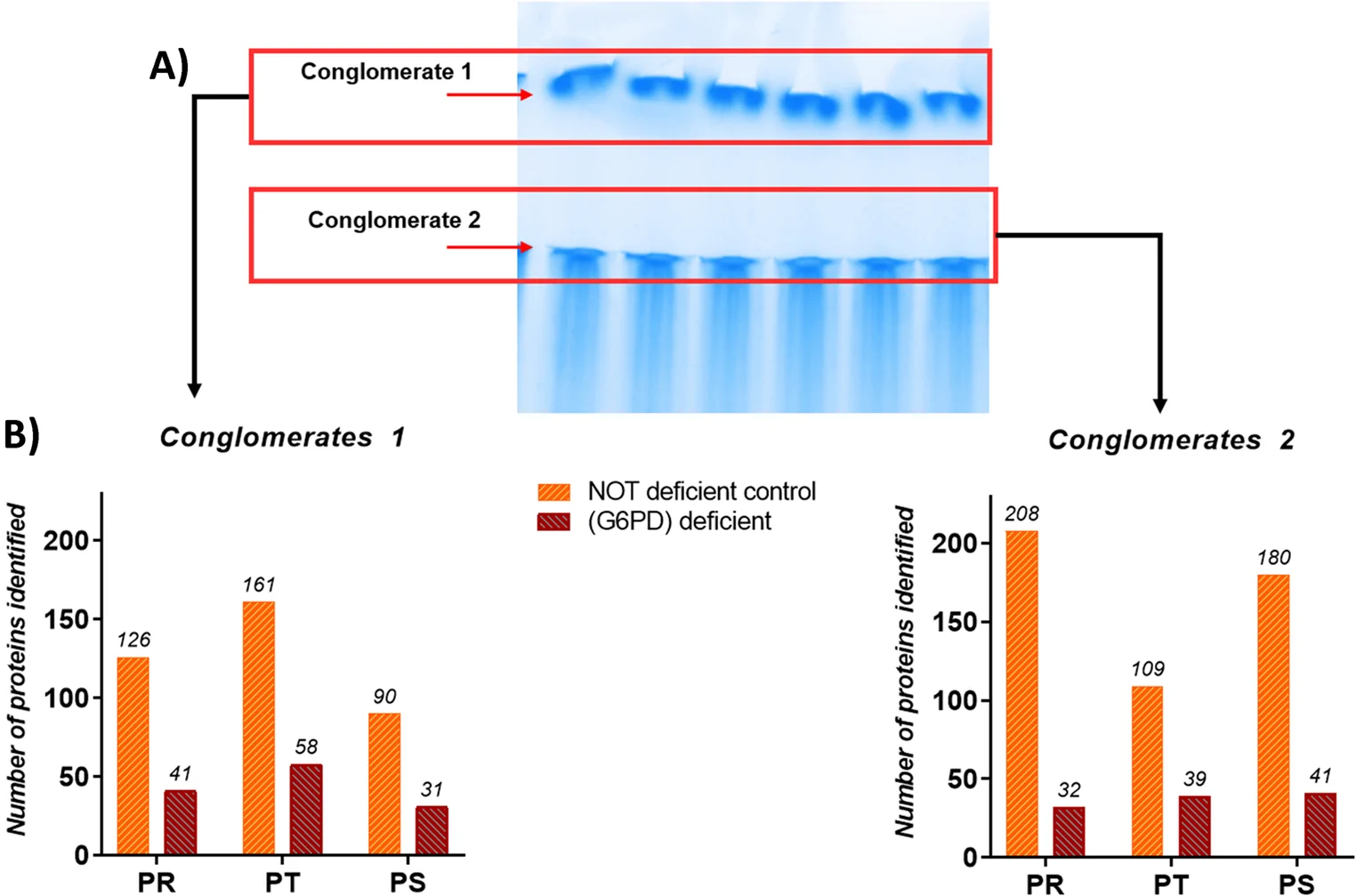
Overview of the isolation and identification of *Plasmodium falciparum* proteins from conglomerate bands**.** (A) Blue-native electrophoresis of infected erythrocyte membrane protein complexes derived from not deficient control and G6PD-deficient erythrocytes infected with *Plasmodium falciparum*. A total of 200 µg of solubilized protein was separated using a gel comprising a 3% stacking layer and an 8% resolving layer. (B) The number of proteins identified within each protein conglomerate 1 and 2 across different developmental stages are indicated for ring (PR, Proteins in Ring stage), trophozoites (PT) and schizont (PS)

The Blue-Native electropherograms exhibited limited resolution; however, two distinct bands representing the highest molecular weight fractions were consistently observed across all analyzed samples. The first was retained at the entrance to the stacking gel, while the second was resolved at the entrance of the resolving gel (Fig. 2a). They were designated as conglomerate 1 and 2, respectively for ring, trophozoite and schizont stages. These bands underwent tryptic digestion and were subsequently analyzed via liquid chromatography coupled with high-resolution mass spectrometry (LC-MS) on the EvoSep One - timsTOF platform. The raw data were processed using the FragPipe platform, incorporating the *Plasmodium falciparum* proteome database from UniProt. A comparative analysis of the protein content within these conglomerates was conducted (Fig. 2-b) for enzymatically healthy and G6PD-deficient erythrocytes infected with *Plasmodium falciparum.* The analysis was paired by band and stage; that is, proteins from conglomerate 1 identified in rings for G6PD deficient erythrocytes were compared exclusively with those identified in the conglomerate 1 of the ring phase in control (non-deficient) erythrocytes. The same was applied to proteins identified at trophozoites and schizonts. To ensure robustness, only proteins identified in both biological replicates for each condition were considered for further analysis.

**Fig. 3:**
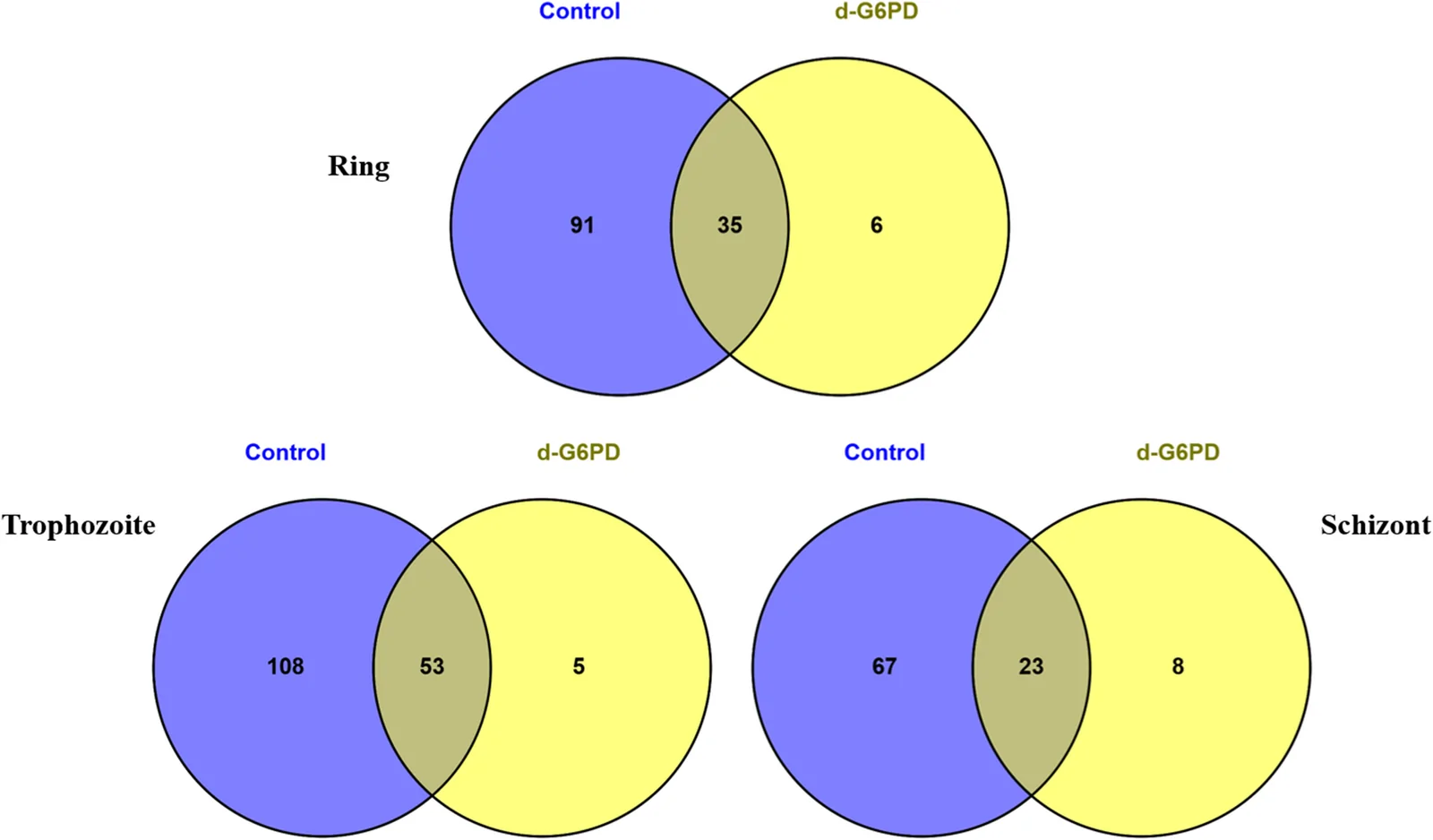
Overlap analysis of *Plasmodium falciparum* proteins identified in the conglomerate 1. The data presented in the graphs correspond to proteins identified across asexual life cycle of the parasite in conglomerate 1 for each stage. Two biological replicates from non-deficient and G6PD-deficient erythrocytes were used

In Protein conglomerate 1 (Fig. 3), a total of 126 *P. falciparum* proteins were identified in the non-deficient control samples during the ring stage, whereas only 41 proteins were identified in the G6PD-deficient samples, with 35 proteins common to both conditions. This pattern persisted across both trophozoite and schizont stages (Fig. 3), where a greater number of *Plasmodium* proteins were identified in non-deficient controls. Among the G6PD-deficient erythrocytes, the schizont stage showed the lowest number of identified *Plasmodium* proteins.

Globally, the total number of *P. falciparum* proteins identified in G6PD-deficient erythrocyte membranes represented 32.5%, 36%, and 34.4% of those identified in the non-deficient erythrocytes during the ring, trophozoite, and schizont stages, respectively.

**Fig. 4:**
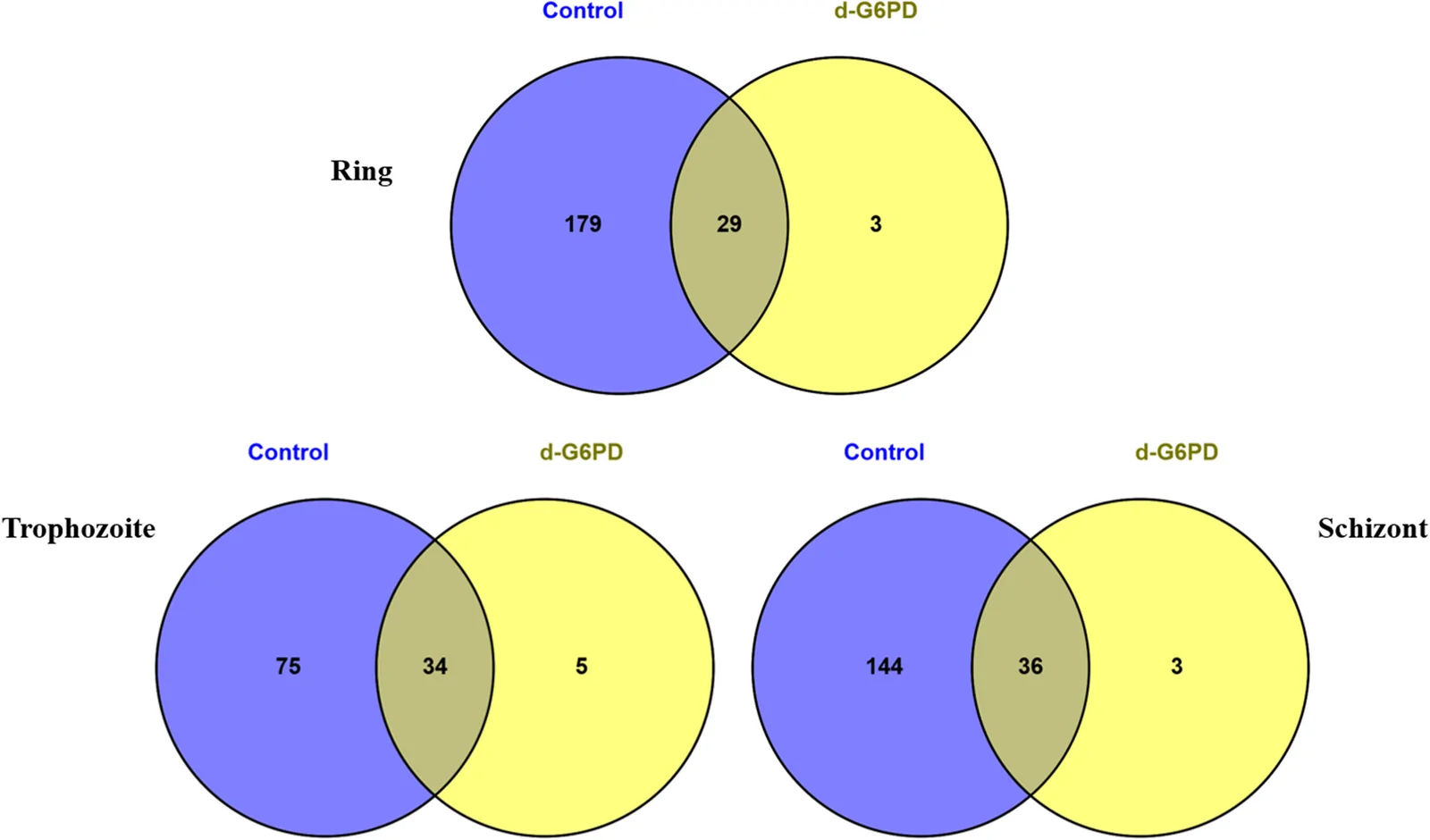
Overlap analysis of *Plasmodium falciparum* proteins identified in the conglomerate 2. The data presented in the graphs correspond to proteins identified across asexual life cycle of the parasite in conglomerate 2 for each stage. Two biological replicates from non-deficient and G6PD-deficient erythrocytes were used

A similar behavior was observed for conglomerate 2 (Fig. 4). The proportion of *Plasmodium* proteins identified in G6PD-deficient erythrocytes was approximately 15.3%, 39% and 21% of those identified in non-deficient controls during the ring, trophozoite and schizont stages, respectively.

**Fig. 5:**
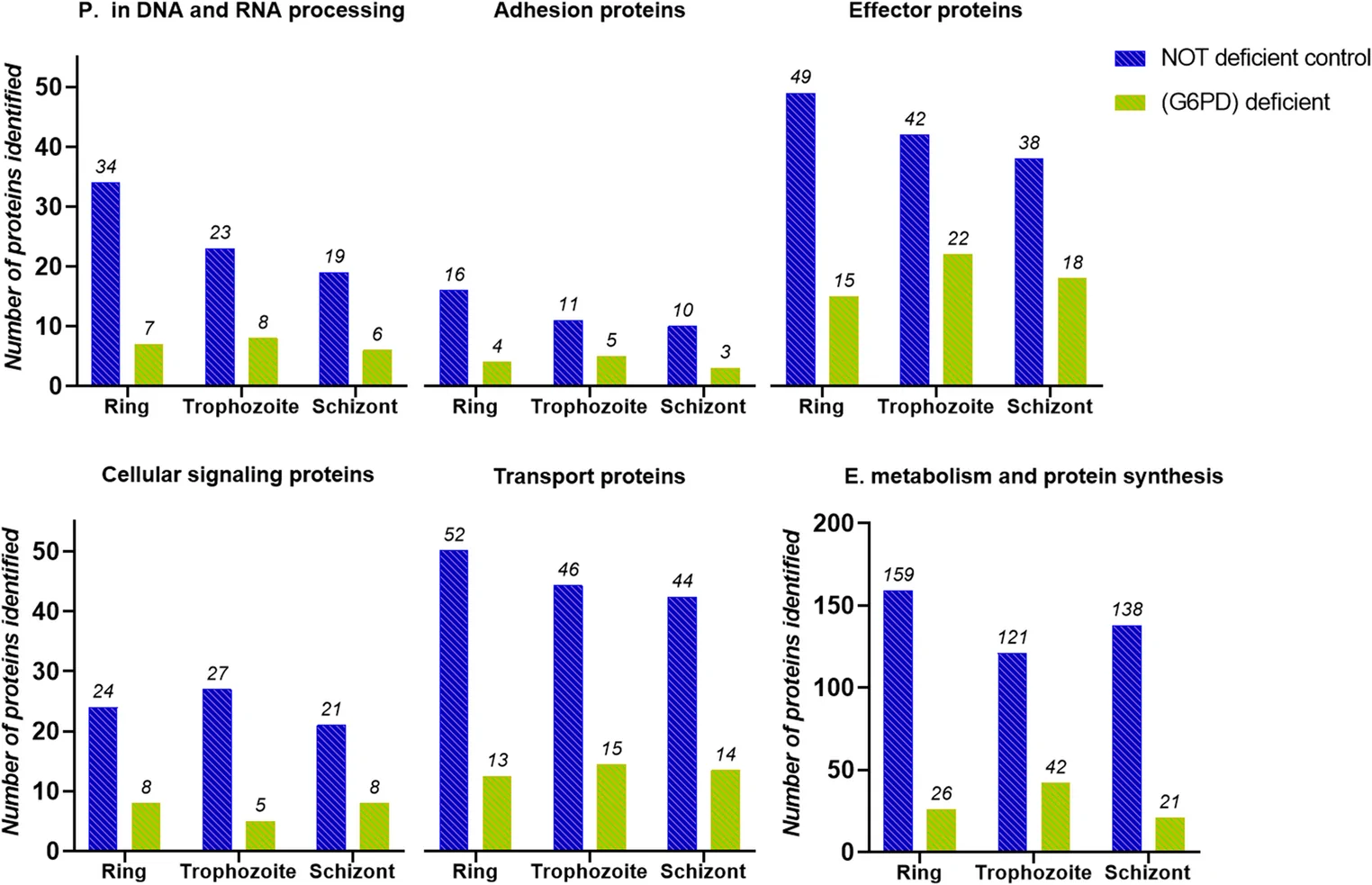
*Plasmodium falciparum* proteins categorized by functional roles and intraerythrocytic developmental stages. *Plasmodium falciparum* proteins identified were clustered into six functional classes. The panels show the number of proteins identified in the ring, trophozoite, and schizont stages, comparing non-deficient control erythrocytes (blue bars) with G6PD-deficient erythrocytes (green bars). A consistent reduction in the number of proteins identified in the context of G6PD deficiency is evident, particularly within the effector and metabolic categories

The identified proteins were classified according to their primary biological functions, allowing a functional overview of the *Plasmodium falciparum* exportome during intraerythrocytic development (Fig. 5). Proteins differentially identified between the control and G6PD-deficient groups are listed in Tables 1 and 2, while the complete list of identified proteins is provided in supplementary tables S1 to S2. Six major functional categories were defined: (1) effector proteins, which encompass key proteins responsible for remodeling and maintaining the structural integrity of the host erythrocyte; (2) adhesion proteins, which mediate interactions between the parasite and the erythrocyte membrane; (3) transport proteins, responsible for the trafficking and export of parasite-derived proteins to the host cytosol or membrane; (4) signaling proteins, which modulate biochemical pathways in response to stimuli within the host cell; (5) proteins associated with energy metabolism and protein synthesis, including glycolytic enzymes and components of the parasite’s translational machinery; and (6) proteins involved in DNA and RNA processing, which ensure the accurate transcription, replication, and maintenance of the parasite’s genetic material. This classification provides a comprehensive framework for assessing the functional impact of G6PD deficiency on biological processes essential for *P. falciparum* survival and the manifestation of severe symptoms of malaria.

**Table 1:** Plasmodium falciparum proteins differentially identified on the membrane fraction of infected erythrocytes from conglomerate 1

Conglomerate 1 - Effector proteins				
Stage	ID	Protein	Condition	
			Control	d-G6PD
R, T	CADF1_PLAF7	Cofilin/actin-depolymerizing factor homolog 1	**+**	**-**
R, S	ENO_PLAF7	Enolase	**+**	**-**
R, S	Q8IEU2_PLAF7	Gamete antigen 27/25	**+**	**-**
R, T	GAP50_PLAF7	Glideosome-associated protein 50	**+**	**-**
R, T,S	Q8IIJ8_PLAF7	Heat shock protein 101	**+**	**-**
R, S	Q8IKM6_PLAF7	Inner membrane complex sub-compartment protein 3	**+**	**-**
R, T,S	Q8I3L4_PLAF7	Long-chain-fatty-acid–CoA ligase	**+**	**-**
R, S	Q8IFM0_PLAF7	Plasmodium RESA N-terminal domain-containing protein	**+**	**-**
R, T	Q8I485_PLAF7	Rhoptry-associated protein 3	**+**	**-**
T, S	Q8I4R5_PLAF7	Rhoptry neck protein 3	**+**	-
*Conglomerate 1 - Adhesion proteins*				
R, S	O77310_PLAF7	Cytoadherence linked asexual protein 3.1	**+**	**-**
R, T	Q8I0U8_PLAF7	Merozoite surface protein 1	**+**	**-**
*Conglomerate 1 - Transport proteins*				
R, T,S	Q8IIF0_PLAF7	Exported protein 1	**+**	**-**
R, T,S	A0A5K1K8H7_PLAF7	Ras-related protein Rab-1B	**+**	**-**
R, S	Q8IAQ8_PLAF7	V-type proton ATPase subunit a, putative	**+**	**-**
R, T	A0A5K1K967_PLAF7	Elongation factor 1-gamma, putative	**+**	**-**
R, T	Q8IKW5_PLAF7	Elongation factor 2	**+**	**-**
T, S	Q8IEI6_PLAF7	EMP1-trafficking protein	**+**	-
*Conglomerate 1 - Cellular signaling proteins*				
R, T	Q7KQK0_PLAF7	cAMP-dependent protein kinase regulatory subunit	**+**	**-**
R, T	Q8IJ74_PLAF7	Haloacid dehalogenase-like hydrolase	**+**	**-**
R, T,S	Q8IB31_PLAF7	Ubiquitin-like domain-containing protein	**+**	**-**
R, S	Q8IBA0_PLAF7	Receptor for activated c kinase	**+**	**-**
T, S	Q8IIR9_PLAF7	Casein kinase 2, alpha subunit	**+**	-
*Conglomerate 1 - Proteins involved in DNA and RNA processing*				
R, T	O97320_PLAF7	Histone H2A	**+**	**-**
R, S	PGK_PLAF7	Phosphoglycerate kinase	**+**	**-**
R, T,S	Q8IE67_PLAF7	Phosphoribosylpyrophosphate synthetase	**+**	**-**
R, S	Q8I2Z8_PLAF7	Probable ATP-dependent 6-phosphofructokinase	**+**	**-**
R, T	Q8IJX3_PLAF7	Single-strand telomeric DNA-binding protein GBP2, putative	**+**	**-**
R, S	TBB_PLAF7	Tubulin beta chain	**+**	**-**
*Conglomerate 1 - Energy metabolism and protein synthesis*				
R, T,S	Q8IJW0_PLAF7	26 S protease regulatory subunit 4, putative	**+**	**-**
R, T,S	Q8IAX5_PLAF7	40 S ribosomal protein S16, putative	**+**	**-**
R, T,S	Q8IFP2_PLAF7	40 S ribosomal protein S19	**+**	**-**
R, T,S	Q8ILE8_PLAF7	60 S ribosomal protein L14, putative	**+**	**-**
R, T,S	Q8IE85_PLAF7	60 S ribosomal protein L6, putative	**+**	**-**
R, T,S	Q8IJ34_PLAF7	ADP/ATP translocase	**+**	**-**
R, T,S	Q8I2W3_PLAF7	Nucleosome assembly protein	**+**	**-**

Summary table presenting the differentially identified proteins in conglomerate 1 across two or more infection stages (*R*= ring, *T*= trophozoite, and *S*= schizont) in G6PD-deficient and non-deficient erythrocytes infected by *Plasmodium falciparum*. The table includes the protein identification (ID). The condition column indicates whether the protein was identified in non-deficient (Controls) or G6PD-deficient donors (d-G6PD), with a (+) presence and a (-) absence

**Table 2:** Plasmodium falciparum proteins differentially identified on the membrane fraction of infected erythrocytes from conglomerate 2

Conglomerate 2 - Effector proteins				
Stage	ID	Protein	Condition	
			Control	d-G6PD
R, S	Q8I2G1_PLAF7	Ring-exported protein 1	+	**-**
R, S	Q8I3L4_PLAF7	Long-chain-fatty-acid–CoA ligase	+	**-**
R, S	Q8IC05_PLAF7	Heat shock protein 90	+	**-**
R, S	Q8III6_PLAF7	Heat shock protein 90, putative	+	**-**
R, S	Q8IIJ8_PLAF7	Heat shock protein 101	+	**-**
R, S	Q8IIV8_PLAF7	Thioredoxin-like mero protein	+	**-**
R, S	Q8IL88_PLAF7	HSP40, subfamily A	+	**-**
*Conglomerate 2 - Adhesion proteins*				
R, T,S	O77309_PLAF7	Cytoadherence linked asexual protein 3.2	+	**-**
R, T,S	P113_PLAF7	Surface protein P113	+	**-**
R, T,S	Q8I0U8_PLAF7	Merozoite surface protein 1	+	**-**
R, S	Q8I2B4_PLAF7	Erythrocyte binding antigen-181	+	**-**
R, S	RH1_PLAF7	Reticulocyte-binding protein homolog 1	+	**-**
*Conglomerate 2 - Transport proteins*				
R, S	Q8IJ76_PLAF7	Early transcribed membrane protein 10.2	+	**-**
R, S	Q8IDB8_PLAF7	YOP1-like protein, putative	+	**-**
R, T,S	ARF1_PLAF7	ADP-ribosylation factor 1	+	**-**
R, S	Q8I298_PLAF7	StAR-related lipid transfer protein	+	**-**
R, T,S	Q8I305_PLAF7	Major facilitator superfamily-related transporter, putative	+	**-**
R, S	Q8I5A9_PLAF7	Ras-related protein Rab-2	+	**-**
R, S	Q8I5M3_PLAF7	Dynamin-like protein, putative	+	**-**
R, T,S	Q8I6Z4_PLAF7	ABC transporter E family member 1, putative	+	**-**
R, S	Q8IHR4_PLAF7	Dynamin-like protein	+	**-**
R, T,S	Q8IIU7_PLAF7	Translocon component PTEX88	+	**-**
R, S	VATA_PLAF7	V-type proton ATPase catalytic subunit A	+	**-**
R, T	Q8IKJ0_PLAF7	V-type proton ATPase subunit	+	**-**
T, S	O77389_PLAF7	Formate-nitrite transporter	+	**-**
T, S	Q7KWJ5_PLAF7	Hexose transporter	+	**-**
R, S	6Q76NM4_PLAF7	Ras-related protein Rab-11 A	+	**-**
*Conglomerate 2 - Cellular signaling proteins*				
R, S	THIO2_PLAF7	Thioredoxin 2	+	**-**
R, T,S	1433I_PLAF7	14-3-3 protein I	+	**-**
R, S	Q7KQK6_PLAF7	GTP-binding nuclear protein	+	**-**
R, S	C6KT34_PLAF7	Cell division cycle protein 48 homologue, putative	+	**-**
R, S	Q8II87_PLAF7	CRAL/TRIO domain-containing protein, putative	+	**-**
T, S	Q8IDH5_PLAF7	Thioredoxin-related protein, putative	+	**-**
R, S	Q8IKJ1_PLAF7	Serine/threonine protein phosphatase UIS2, putative	+	**-**
*Conglomerate 2 - Proteins involved in DNA and RNA processing*				
R, S	C6KSV9_PLAF7	SWIB/MDM2 domain-containing protein	+	**-**
R, S	Q6ZLZ9_PLAF7	Tubulin alpha chain	+	**-**
R, T,S	Q8IE67_PLAF7	Phosphoribosylpyrophosphate synthetase	+	**-**
R, T,S	Q8II73_PLAF7	Spermidine synthase	+	**-**
R, S	Q8IIW0_PLAF7	Chromatin remodeling protein	+	**-**
R, S	TBB_PLAF7	Tubulin beta chain	+	**-**
*Conglomerate 2 - Energy metabolism and protein synthesis*				
R, T,S	Q8IEQ1_PLAF7	26 S protease regulatory subunit 10B, putative	+	**-**
R, S	Q8IKH3_PLAF7	26 S proteasome regulatory subunit RPN2, putative	+	**-**
R, T,S	Q8IAX5_PLAF7	40 S ribosomal protein S16, putative	+	**-**
R, T,S	Q8IM10_PLAF7	40 S ribosomal protein S8	+	**-**
R, T,S	Q8I3R0_PLAF7	40 S ribosomal protein S9, putative	+	**-**
R, T,S	C6KSY6_PLAF7	60 S ribosomal protein L19	+	**-**
R, T,S	Q8I3T9_PLAF7	60 S ribosomal protein L2	+	**-**
R, T,S	Q8ILK3_PLAF7	60 S ribosomal protein L21	+	**-**
R, T,S	Q8IKK7_PLAF7	Glyceraldehyde-3-phosphate dehydrogenase	+	**-**
R, S	O97245_PLAF7	Glycerol-3-phosphate dehydrogenase	+	**-**
R, T	PLM3_PLAF7	Plasmepsin III	+	**-**
R, S	PLM4_PLAF7	Plasmepsin IV	+	**-**
R, S	Q8IDG2_PLAF7	Proteasome subunit alpha type	+	**-**

Summary table presenting the differentially identified proteins in conglomerate 2 across two or more infection stages (*R*= ring, *T*= trophozoite, and *S*= schizont) in G6PD-deficient and non-deficient erythrocytes infected by *Plasmodium falciparum*. The table includes the protein identification (ID). The condition column indicates whether the protein was identified in non-deficient (Controls) or G6PD-deficient donors (d-G6PD), with a (+) presence and a (-) absence

The potential effects of this reduction on the surface area of the G6PD deficient erythrocyte are discussed in the next section.

## Discussion

Proteomic analysis performed on protein conglomerates 1 and 2 in both non-deficient controls and G6PD-deficient erythrocytes, across the three intraerythrocytic stages of *P. falciparum*, suggest that this polymorphism exerts a protective effect mediated by significantly reducing the localization of parasite exportome proteins to the host cell membrane. Parasites developing in healthy erythrocytes exhibited a broad repertoire of proteins reported to be necessary for the growth, survival, and virulence of the pathogen. In contrast, those in G6PD-deficient erythrocytes exhibited a restricted landscape, predominantly limited to proteins associated with immediate survival and stress response.

It is essential to emphasize that the absence of the *P. falciparum* proteins does not unequivocally indicate its nonexistence within the parasite (Tables 1 and 2). Rather, it may suggest that these proteins either not being exported or incorporated efficiently into the membrane under conditions of glucose-6-phosphate dehydrogenase (G6PD) deficiency. Alternatively, they may be present at lower levels that fall below the sensitivity threshold of the proteomic approach employed. Likewise, due a reduced number of biological samples included per group, inferential statistical analysis was not considered. Despite these limitations, the results reveal a substantial reduction in the proteins exported by the parasite to the surface of the G6PD-deficient infected erythrocyte membrane.

This observed behavior is consistent with several of the protective mechanisms previously proposed for G6PD deficiency; most significantly, it parallels research indicating that G6PD deficiency confers a protective effect against severe malaria by decreasing essential parasite survival mechanisms such as cytoadherence and autoagglutination (Batinovic et al. 2017; Gabriela et al. 2022; Liang et al. 2020).

The absence or diminution of effector proteins, including RESA, heat shock protein 90 and 101, and rhoptry-associated proteins, may play a significant role in the development of tolerance mechanisms against severe malaria. This assertion is reinforced by the critical function of RAP3 in mediating adhesion and facilitating the formation of the parasitophorous vacuole during red blood cell invasion (Counihan et al. 2013). Furthermore, a decrease in the number of essential heat shock proteins can also compromise the export of virulence factors and threaten the survival of the parasite by affecting different processes. In the case of heat shock protein 101, a component of the PTEX translocator, the unfolding of parasite proteins destined for export would be affected (Elsworth et al. 2014); while heat shock protein 90, being related to thermal or oxidative stress, could negatively affect the correct folding of the parasite’s exported proteins (Kats et al. 2015).

Delayed detection of the Ring-Infected Erythrocyte Surface Antigen (RESA) was also observed. In the control group, this antigen was identified during the ring stage; however, in cases of d-G6PD, it was detected only from the trophozoite stage, suggesting a potential delay in RESA synthesis and function. RESA associates with the erythrocyte membrane, stabilizing the host cell by interacting with cytoskeletal proteins, such as spectrins; thereby enhancing cellular rigidity and facilitating cytoadherence of infected erythrocytes (Pei et al. 2007). Furthermore, RESA may modulate cytokine production, including tumor necrosis factor (TNF), which is critical to the inflammatory response during malaria. Evidence suggests that RESA induces TNF production in human macrophages, underscoring its role in modulating the host immune response (Picot et al. 1993).

Additionally, proteins essential for the parasite adhesion and its interactions with the host play a crucial role in the in vivo survival and virulence of the parasite. Proteins such as CLAG 3.1 and CLAG 3.2 (cytoadhesion-linked asexual proteins), have been found to be differentially found in non-deficient controls. CLAG and its associated proteins are integral components of the RhopH complex (Pasternak et al. 2022), which serves two primary functions: first, it facilitates the entry of merozoites into host cells (Ito et al. 2017); and second, it is incorporated into the erythrocyte membrane to increase solute permeability through the formation of new permeability pathways (NPP). These pathways are critical for nutrient uptake via surface anion channels (Desai 2014; Gupta et al. 2018).

Furthermore, previous studies have also demonstrated that G6PD-deficient erythrocytes, upon infection with *Plasmodium falciparum*, are more efficiently eliminated by phagocytosis during the early stages of infection (specifically the ring stage). This premature clearance diminishes the parasitemia of mature forms (trophozoites and schizonts), thereby mitigating the severity of the disease (Ayi et al. 2004; Cappadoro et al. 1998). In this context, the reduction in adhesion proteins observed in the membrane ghosts of G6PD-deficient erythrocytes may also lead to decreased rosette formation and impaired sequestration within blood vessels.

Similarly, a differential reduction in proteins implicated in protein transport was noted, particularly regarding the EMP1 trafficking protein, Exported Protein 1 (EXP1), Ras-related protein (Ras-1B), and the translocon component PTEX 88. The EMP1 trafficking protein is crucial for the intracellular transport of the virulence antigen *Pf*EMP1 to the host cell membrane, thereby facilitating the adhesion of infected red blood cells to the vascular endothelium (McHugh et al. 2020). EXP1 is implicated in the biogenesis of the parasitophorous vacuole, while various Rab GTPase proteins play key roles in the regulation of vesicular trafficking. Additionally, components of the auxiliary PTEX88 complex were found to be absent. In an in vivo study utilizing *Plasmodium falciparum 3D7* and *Plasmodium berghei* with PTEX88 knockdown, Scott et al. reported a decreased capacity of the parasite to adhere to peripheral tissues, which correlated with reduced adherence of infected erythrocytes to the endothelial receptor CD36 (Chisholm et al. 2016). This is consistent with observations in G6PD-deficient erythrocytes infected by *Plasmodium*, which exhibit minimal knob formation and significantly reduced endothelial adhesion, ultimately leading to their clearance by the spleen (Palasuwan et al. 2022). The decrease in PTEX88 may be directly or indirectly associated with the diminished cytoadherence observed in G6PD-deficient infected erythrocytes, suggesting a disruption in the virulence mechanisms of the parasite.

The variety of signaling proteins identified was more limited in G6PD-deficient erythrocytes. Key components in this context include classical kinases, phosphatases, and peroxiredoxins. Kinases phosphorylate ubiquitin ligase, thereby regulating the export of the 26 S proteasome from the parasitophorous vesicle to the erythrocyte cytoplasm. This phosphorylation enhances the ubiquitination of α-spectrin, which subsequently triggers the remodeling of the host spectrin-actin network (Zheng et al. 2024). Furthermore, the 14-3-3 protein I, which was diminished in G6PD deficient erythrocytes infected with the parasite, functions as an adaptor protein that serves as a scaffold, linking the regulatory subunit of Protein Kinase A (PKA) with Calcium-Dependent Protein Kinase 1 (CDPK1). This interaction is critical for the regulating signaling events associated with merozoite egress and exit (Jain et al. 2020; More et al. 2020).

The decrease in this protein may disrupt the complex and the intracellular signaling pathways essential for both parasite egress and invasion. Lastly, the reduction of the CRAL/TRIO domain-containing protein, which is implicated in lipid transport, may compromise membrane integrity or flexibility, thereby adversely affecting cellular growth and division (Šťastný et al. 2025).

The number of proteins involved in energy metabolism and protein synthesis identified in G6PD-deficient cells was also lesser than non-deficient. This suggests an affectation in functions such as protein synthesis, ATP production, and replication or its ability to maintain protein homeostasis (Briquet et al. 2018; Hsu et al. 2023). Likewise, several proteins required for replication and subtelomeric gene expression were affected (Edwards-Smallbone et al. 2022), alongside structural proteins such as tubulin beta chain and enzymes such as phosphoribosylpyrophosphate synthetase. Together, the reduction of these proteins could disrupt mitotic processes and parasite proliferation (Chakrabarti et al. 2021; Zhang et al. 2017).

Despite the significant reduction in the quantity of exported proteins, our observations indicate that the parasite can still replicate effectively in G6PD-deficient erythrocytes under in vitro conditions. This finding aligns with previous studies demonstrating that various *Plasmodium* strains maintain normal invasion and growth rates in G6PD-deficient erythrocytes in vitro (Ayi et al. 2004). This suggests that many of the proteins exported to the host cell membrane are not critical for short-term replication under laboratory conditions, where a host immune response and splenic clearance are absent. Consequently, the parasite can survive with a diminished remodeling of the host cell membrane. However, within the human host, this scenario would likely enhance the clearance of infected erythrocytes and better constrain the progression of the infection.

This set of results provides a starting point for a comprehensive understanding of the potential metabolic alterations occurring in G6PD-deficient erythrocytes infected with *Plasmodium falciparum*, which directly influence the parasite’s exportome. Consequently, the reduction in proteins associated with G6PD deficiency in infected erythrocytes suggests that the protozoan may be unable to fully remodel the host cell and expose its virulence factors, thereby reducing its pathogenicity. These alterations are consistent with the established protective role of this erythroenzymopathy against severe malaria. Thus, under conditions of heightened oxidative stress, attributable to the decreased antioxidant capacity of G6PD-deficient erythrocytes, *P. falciparum* appears to prioritize the expression of proteins essential for fundamental cellular maintenance, such as those involved in translation and central metabolism. This prioritization occurs at the expense of proteins required for effectively remodeling the architecture of the host erythrocyte surface, facilitating surface adhesion, evading the immune response, and invading new cells. Finally, our findings may illuminate new routes for investigating the complex and ancient relationship between the parasite and host in human malaria.

A significant limitation of this study is the limited number of biological samples included per group. Consequently, the current research was not structured to facilitate inferential statistical comparisons. The proteomic differences identified should, therefore, be interpreted as exploratory patterns of protein detection rather than as statistically validated changes in differential abundance. Future investigations involving larger cohorts and independent biological replicates will be necessary to corroborate these observations.

## Conclusion

The alterations in *Plasmodium falciparum* exported proteins on the membrane of G6PD-deficient erythrocytes suggests that this polymorphism creates a hostile environment for the export and/or anchoring of parasite proteins to the host cell membrane. This modification in the *Plasmodium* exportome may partially elucidate the protective mechanisms against severe malaria observed in G6PD-deficient individuals, as it compromises the parasite’s capacity to adhere to the host cell and evade the immune response. Our findings suggest a multifactorial protective mechanism wherein G6PD deficiency restricts the remodeling of the erythrocyte surface, thereby facilitating early clearance prior to the onset of severe pathology.

## Supplementary Information

Below is the link to the electronic supplementary material.

Supplementary Material 1 (DOCX 642 KB)

## Data Availability

Our mass spectrometry data have been deposited in the ProteomeXchange Consortium via the PRIDE repository under the identifier PXD061301.

## Abbreviations

RBC: Red blood cell
d-G6PD: glucose-6-phosphate dehydrogenase deficiency
PfEMP1: P. falciparum erythrocyte membrane protein 1
LC-MS-tims-TOF: Liquid chromatography–mass spectrometry coupled to trapped ion mobility spectrometry and time-of-flight mass analyzer
