# Supplementary material for: G6PD deficiency and the exportome of plasmodium falciparum FCB-2: A comparative analysis utilizing one-dimensional blue-native electrophoresis and timstof mass spectrometry
Source: Parasitol Res. 2026 Jul 2;125(1):113. doi: 10.1007/s00436-026-08717-y (PMC13612704; doi:10.1007/s00436-026-08717-y)
Supplement: Supplementary file 1 [file 436_2026_8717_MOESM1_ESM.docx]

**Supplementary Information**

**G6PD Deficiency and the Exportome of *Plasmodium falciparum* FCB-2: A Comparative Analysis Utilizing One-Dimensional Blue-Native Electrophoresis and timsTOF Mass Spectrometry**

**Authors**

Isaac. De la Rosa Cogollo ^a^, María. Luisa Hernáez ^b^, Luis. Felipe Clemente ^b^, Albeiro. Marrugo-Padilla ^c^, Erika. Rodríguez-Cavallo ^c*^ and Darío. Méndez-Cuadro ^a*^

**Affiliations**: ^a^ University of Cartagena, Faculty of Exact and Natural Sciences, Analytical Chemistry and Biomedicine Group, Cartagena de Indias, Colombia.

^b^ Complutense University of Madrid (UCM), Proteomics Unit, Faculty of Pharmacy, Madrid, Spain.

^c^ University of Cartagena, Faculty of Pharmaceutical Sciences, Analytical Chemistry and Biomedicine Group, Cartagena de Indias, Colombia.

***Figure S1.*** SDS-PAGE electrophoresis of erythrocyte membrane proteins infected by *Plasmodium falciparum*. This analysis aimed to validate the extraction of protein ghosts.

**Supplementary** **table 1.** Protein identified in conglomerate 1 of erythrocytes with the ring forms of *P. falciparum FCB-2.*

| ***Conglomerate 1 Ring*** | | | | |
| --- | --- | --- | --- | --- |
| ***N°*** | ***ID*** | ***Protein name*** | ***Condition*** | |
|  |  |  | ***Control*** | ***d-G6PD*** |
| ***Proteins involved in DNA and RNA processing*** | | | | |
| 1 | C6KT34_PLAF7 | Cell division cycle protein 48 homologue, putative | **-** | **+** |
| 2 | O97320_PLAF7 | Histone H2A | **+** | **-** |
| 3 | Q8IIV1_PLAF7 | Histone H2B | **+** | **+** |
| 4 | C6KSV0_PLAF7 | Histone H3 | **-** | **+** |
| 5 | Q8IIV2_PLAF7 | Histone H4 | **+** | **+** |
| 6 | PGK_PLAF7 | Phosphoglycerate kinase | **+** | **-** |
| 7 | Q8IE67_PLAF7 | Phosphoribosylpyrophosphate synthetase | **+** | **-** |
| 8 | Q8IJS7_PLAF7 | PRE-binding protein | **+** | **-** |
| 9 | Q8I2Z8_PLAF7 | Probable ATP-dependent 6-phosphofructokinase | **+** | **-** |
| 10 | C6KTA4_PLAF7 | Pyruvate kinase | **+** | **-** |
| 11 | Q8IJX3_PLAF7 | Single-strand telomeric DNA-binding protein GBP2, putative | **+** | **-** |
| 12 | Q8II73_PLAF7 | Spermidine synthase | **+** | **-** |
| 13 | Q6ZLZ9_PLAF7 | Tubulin alpha chain | **+** | **+** |
| 14 | TBB_PLAF7 | Tubulin beta chain | **+** | **-** |
| ***Adhesion proteins*** | | | | |
| 15 | O77310_PLAF7 | Cytoadherence linked asexual protein 3.1 | **+** | **-** |
| 16 | O77309_PLAF7 | Cytoadherence linked asexual protein 3.2 | **+** | **-** |
| 17 | Q8IAK1_PLAF7 | Erythrocyte membrane protein 1, PfEMP1 | **+** | **+** |
| 18 | Q8I0U8_PLAF7 | Merozoite surface protein 1 | **+** | **-** |
| ***Effector proteins*** | | | | |
| 19 | ACT1_PLAF7 | Actin-1 | **+** | **+** |
| 20 | ACT2_PLAF7 | Actin-2 | **+** | **-** |
| 21 | Q8IJR6_PLAF7 | Autophagy-related protein 18 | **+** | **-** |
| 22 | CADF1_PLAF7 | Cofilin/actin-depolymerizing factor homolog 1 | **+** | **-** |
| 23 | ENO_PLAF7 | Enolase | **+** | **-** |
| 24 | Q8IEU2_PLAF7 | Gamete antigen 27/25 | **+** | **-** |
| 25 | GAP50_PLAF7 | Glideosome-associated protein 50 | **+** | **-** |
| 26 | Q8IIJ8_PLAF7 | Heat shock protein 101 | **+** | **-** |
| 27 | Q8II24_PLAF7 | Heat shock protein 70 | **+** | **+** |
| 28 | Q8IC05_PLAF7 | Heat shock protein 90 | **+** | **+** |
| 29 | C0H571_PLAF7 | High molecular weight rhoptry protein 2 | **+** | **+** |
| 30 | Q8I395_PLAF7 | High molecular weight rhoptry protein 3 | **+** | **-** |
| 31 | Q8IKM6_PLAF7 | Inner membrane complex sub-compartment protein 3 | **+** | **-** |
| 32 | Q8I3L4_PLAF7 | Long-chain-fatty-acid--CoA ligase | **+** | **-** |
| 33 | Q8IDQ9_PLAF7 | Phosphoethanolamine N-methyltransferase | **+** | **+** |
| 34 | Q8IFM0_PLAF7 | Plasmodium RESA N-terminal domain-containing protein | **+** | **-** |
| 35 | Q8I4R5_PLAF7 | Rhoptry neck protein 3 | **+** | **+** |
| 36 | Q8ILZ1_PLAF7 | Rhoptry-associated protein 1 | **+** | **+** |
| 37 | Q8I484_PLAF7 | Rhoptry-associated protein 2 | **+** | **+** |
| 38 | Q8I485_PLAF7 | Rhoptry-associated protein 3 | **+** | **-** |
| 39 | Q8I0U6_PLAF7 | Ring-infected erythrocyte surface antigen | **+** | **-** |
| ***Cellular signaling proteins*** | | | | |
| 40 | 1433I_PLAF7 | 14-3-3 protein I | **+** | **+** |
| 41 | Q7KQK0_PLAF7 | cAMP-dependent protein kinase regulatory subunit | **+** | **-** |
| 42 | Q7KQK6_PLAF7 | GTP-binding nuclear protein | **+** | **+** |
| 43 | Q8IJ74_PLAF7 | Haloacid dehalogenase-like hydrolase | **+** | **-** |
| 44 | Q7KQK2_PLAF7 | Polyubiquitin | **+** | **+** |
| 45 | Q8IL80_PLAF7 | Thioredoxin peroxidase 1 | **+** | **+** |
| 46 | Q8IDH5_PLAF7 | Thioredoxin-related protein, putative | **+** | **-** |
| 47 | Q8IB31_PLAF7 | Ubiquitin-like domain-containing protein | **+** | **-** |
| 48 | Q8IBA0_PLAF7 | Receptor for activated c kinase | **+** | **-** |
| ***Energy metabolism and protein synthesis*** | | | | |
| 49 | Q8I5S6_PLAF7 | Eukaryotic translation initiation factor 3 subunit A | **+** | **-** |
| 50 | Q8I3T1_PLAF7 | Eukaryotic translation initiation factor 3 subunit B | **+** | **-** |
| 51 | ALF_PLAF7 | Fructose-bisphosphate aldolase | **+** | **-** |
| 52 | Q8ILT0_PLAF7 | Glutamate dehydrogenase | **+** | **-** |
| 53 | Q8IJW0_PLAF7 | 26S protease regulatory subunit 4, putative | **+** | **-** |
| 54 | Q8I4U5_PLAF7 | 26S protease regulatory subunit 8, putative | **+** | **-** |
| 55 | Q8IAX5_PLAF7 | 40S ribosomal protein S16, putative | **+** | **-** |
| 56 | Q8IFP2_PLAF7 | 40S ribosomal protein S19 | **+** | **-** |
| 57 | O97248_PLAF7 | 40S ribosomal protein S23, putative | **+** | **-** |
| 58 | Q8IKH8_PLAF7 | 40S ribosomal protein S3 | **+** | **+** |
| 59 | Q8IL02_PLAF7 | 40S ribosomal protein S5 | **+** | **-** |
| 60 | Q8IBN5_PLAF7 | 40S ribosomal protein S5, putative | **+** | **-** |
| 61 | Q8IET7_PLAF7 | 40S ribosomal protein S7 | **+** | **-** |
| 62 | Q8IM10_PLAF7 | 40S ribosomal protein S8 | **+** | **-** |
| 63 | Q8I3R0_PLAF7 | 40S ribosomal protein S9, putative | **+** | **-** |
| 64 | Q8II61_PLAF7 | 60S acidic ribosomal protein P0 | **+** | **-** |
| 65 | Q8ILV2_PLAF7 | 60S ribosomal protein L10, putative | **+** | **-** |
| 66 | Q8IJZ7_PLAF7 | 60S ribosomal protein L13, putative | **+** | **-** |
| 67 | Q8ILE8_PLAF7 | 60S ribosomal protein L14, putative | **+** | **-** |
| 68 | Q8IDI5_PLAF7 | 60S ribosomal protein L17, putative | **+** | **-** |
| 69 | C0H5G3_PLAF7 | 60S ribosomal protein L18-2, putative | **+** | **-** |
| 70 | Q8IDS6_PLAF7 | 60S ribosomal protein L18a | **+** | **-** |
| 71 | Q8I3T9_PLAF7 | 60S ribosomal protein L2 | **+** | **-** |
| 72 | Q8ILK3_PLAF7 | 60S ribosomal protein L21 | **+** | **+** |
| 73 | Q8IEM3_PLAF7 | 60S ribosomal protein L24, putative | **-** | **+** |
| 74 | O77364_PLAF7 | 60S ribosomal protein L26, putative | **+** | **-** |
| 75 | C6KT23_PLAF7 | 60S ribosomal protein L27a, putative | **-** | **+** |
| 76 | Q8IJK8_PLAF7 | 60S ribosomal protein L30e, putative | **+** | **-** |
| 77 | Q8IIB4_PLAF7 | 60S ribosomal protein L35ae, putative | **+** | **-** |
| 78 | Q8I431_PLAF7 | 60S ribosomal protein L4 | **+** | **+** |
| 79 | Q8ILL3_PLAF7 | 60S ribosomal protein L5, putative | **+** | **+** |
| 80 | Q8IE85_PLAF7 | 60S ribosomal protein L6, putative | **+** | **-** |
| 81 | O97250_PLAF7 | 60S ribosomal protein L7, putative | **+** | **-** |
| 82 | Q8ILL2_PLAF7 | 60S ribosomal protein L7a | **+** | **-** |
| 83 | Q8I6Z1_PLAF7 | Acyl-CoA synthetase | **+** | **-** |
| 84 | SAHH_PLAF7 | Adenosylhomocysteinase | **+** | **-** |
| 85 | KAD1_PLAF7 | Adenylate kinase 1 | **+** | **-** |
| 86 | Q8IJ34_PLAF7 | ADP/ATP translocase | **+** | **-** |
| 87 | Q8IKP1_PLAF7 | ATP-dependent RNA helicase DBP5 | **-** | **+** |
| 88 | Q76NM3_PLAF7 | L-lactate dehydrogenase | **+** | **+** |
| 89 | Q8III3_PLAF7 | Nucleolar protein 56, putative | **+** | **-** |
| 90 | Q8I2W3_PLAF7 | Nucleosome assembly protein | **+** | **-** |
| 91 | OAT_PLAF7 | Ornithine aminotransferase | **+** | **+** |
| 92 | PLM1_PLAF7 | Plasmepsin I | **+** | **-** |
| 93 | PLM3_PLAF7 | Plasmepsin III | **+** | **-** |
| 94 | PLM4_PLAF7 | Plasmepsin IV | **+** | **-** |
| 95 | Q8I5H4_PLAF7 | Polyadenylate-binding protein | **+** | **-** |
| 96 | Q8I374_PLAF7 | Proteasome activator 28 subunit beta, putative | **+** | **-** |
| 97 | Q8IDG2_PLAF7 | Proteasome subunit alpha type | **+** | **-** |
| 98 | C6KST3_PLAF7 | Proteasome subunit alpha type-2, putative | **+** | **-** |
| 99 | Q8IAR3_PLAF7 | Proteasome subunit alpha type-6, putative | **-** | **+** |
| 100 | Q8IJT1_PLAF7 | Proteasome subunit beta | **+** | **-** |
| 101 | C0H4Y6_PLAF7 | Protein disulfide-isomerase | **+** | **-** |
| 102 | C0H4A6_PLAF7 | Ribosomal protein L15 | **+** | **+** |
| 103 | Q8IKF0_PLAF7 | RNA helicase | **+** | **+** |
| 104 | Q7K6A4_PLAF7 | S-adenosylmethionine synthase | **+** | **+** |
| 105 | RS3A_PLAF7 | Small ribosomal subunit protein eS1 | **+** | **-** |
| 106 | Q8IAX8_PLAF7 | DNA/RNA-binding protein Alba 1 | **+** | **-** |
| 107 | Q8IJX8_PLAF7 | DNA/RNA-binding protein Alba 3 | **+** | **-** |
| 108 | A0A5K1K8Y8_PLAF7 | DNA/RNA-binding protein Alba 4 | **+** | **-** |
| 109 | Q8I546_PLAF7 | DUF4149 domain-containing protein | **+** | **+** |
| 110 | Q8IKK7_PLAF7 | Glyceraldehyde-3-phosphate dehydrogenase | **+** | **+** |
| 111 | Q8I0V4_PLAF7 | Endoplasmin, putative | **+** | **+** |
| ***Transport proteins*** | | | | |
| 112 | ARF1_PLAF7 | ADP-ribosylation factor 1 | **+** | **-** |
| 113 | Q8II36_PLAF7 | Aquaglyceroporin | **+** | **+** |
| 114 | Q8IHR4_PLAF7 | Dynamin-like protein | **+** | **+** |
| 115 | Q8IIF0_PLAF7 | Exported protein 1 | **+** | **-** |
| 116 | Q8IKC8_PLAF7 | Exported protein 2 | **+** | **+** |
| 117 | O77389_PLAF7 | Formate-nitrite transporter | **+** | **+** |
| 118 | Q8IKR1_PLAF7 | H(+)-exporting diphosphatase | **+** | **-** |
| 119 | Q7KWJ5_PLAF7 | Hexose transporter | **+** | **+** |
| 120 | Q8I3M5_PLAF7 | Karyopherin beta | **+** | **-** |
| 121 | Q8I305_PLAF7 | Major facilitator superfamily-related transporter, putative | **+** | **-** |
| 122 | Q7K6A5_PLAF7 | Multidrug resistance protein 1 | **+** | **-** |
| 123 | Q8I2Q0_PLAF7 | Parasitophorous vacuolar protein 5, putative | **+** | **-** |
| 124 | A0A5K1K8H7_PLAF7 | Ras-related protein Rab-1B | **+** | **-** |
| 125 | Q8I5A9_PLAF7 | Ras-related protein Rab-2 | **+** | **-** |
| 126 | Q8IAQ8_PLAF7 | V-type proton ATPase subunit a, putative | **+** | **-** |
| 127 | A0A5K1K7X4_PLAF7 | Early transcribed membrane protein 5 | **+** | **-** |
| 128 | Q8I0V2_PLAF7 | ATP synthase subunit beta | **+** | **+** |
| 129 | Q9TY94_PLAF7 | ATP-dependent RNA helicase UAP56 | **+** | **-** |
| 130 | Q8I0P6_PLAF7 | Elongation factor 1-alpha | **+** | **+** |
| 131 | A0A5K1K967_PLAF7 | Elongation factor 1-gamma, putative | **+** | **-** |
| 132 | Q8IKW5_PLAF7 | Elongation factor 2 | **+** | **-** |

Note: The condition column indicates whether the protein was identified in non-deficient or G6PD-deficient states, or if it was present in both conditions. A (+) signifies presence, while a (-) signifies absence.

**Supplementary** **table 2.** Protein identified in conglomerate 1 of erythrocytes with the trophozoite forms of *P. falciparum FCB-2.*

| ***Conglomerate 1 Trophozoite*** | | | | |
| --- | --- | --- | --- | --- |
| ***N°*** | ***ID*** | ***Proteín name*** | ***Condition*** | |
|  |  |  | ***Control*** | ***d-G6PD*** |
| ***Proteins involved in DNA and RNA processing*** | | | | |
| 1 | O97320_PLAF7 | Histone H2A | **+** | - |
| 2 | Q8IIV1_PLAF7 | Histone H2B | **+** | - |
| 3 | C6KSV0_PLAF7 | Histone H3 | **+** | - |
| 4 | Q8IIV2_PLAF7 | Histone H4 | **+** | **+** |
| 5 | Q8IJS1_PLAF7 | Hypoxanthine phosphoribosyltransferase | **+** | - |
| 6 | PGK_PLAF7 | Phosphoglycerate kinase | **+** | **+** |
| 7 | Q8IE67_PLAF7 | Phosphoribosylpyrophosphate synthetase | **+** | - |
| 8 | Q8IJS7_PLAF7 | PRE-binding protein | **+** | **+** |
| 9 | C6KTA4_PLAF7 | Pyruvate kinase | **+** | **+** |
| 10 | Q8IBA0_PLAF7 | Receptor for activated c kinase | **+** | - |
| 11 | Q8IIU3_PLAF7 | RuvB-like helicase | **+** | - |
| 12 | Q8IJX3_PLAF7 | Single-strand telomeric DNA-binding protein GBP2, putative | **+** | - |
| 13 | Q8II73_PLAF7 | Spermidine synthase | **+** | **+** |
| 14 | Q6ZLZ9_PLAF7 | Tubulin alpha chain | **+** | - |
| 15 | TBB_PLAF7 | Tubulin beta chain | **+** | **+** |
| ***Adhesion proteins*** | | | | |
| 16 | Q8I0U8_PLAF7 | Merozoite surface protein 1 | **+** | - |
| 17 | O77310_PLAF7 | Cytoadherence linked asexual protein 3.1 | **+** | **+** |
| 18 | Q8I2G2_PLAF7 | Cytoadherence linked asexual protein 9 | **+** | **+** |
| ***Effector proteins*** | | | | |
| 19 | ACT1_PLAF7 | Actin-1 | **+** | **+** |
| 20 | CADF1_PLAF7 | Cofilin/actin-depolymerizing factor homolog 1 | **+** | - |
| 21 | Q8IEU2_PLAF7 | Gamete antigen 27/25 | **+** | **+** |
| 22 | Q8IFN1_PLAF7 | Glideosome associated protein with multiple membrane spans 2 | **+** | - |
| 23 | A0A5K1K8J3_PLAF7 | Glideosome-associated connector | **+** | **+** |
| 24 | GAP50_PLAF7 | Glideosome-associated protein 50 | **+** | - |
| 25 | Q8IIJ8_PLAF7 | Heat shock protein 101 | **+** | - |
| 26 | Q8II24_PLAF7 | Heat shock protein 70 | **+** | **+** |
| 27 | Q8IC05_PLAF7 | Heat shock protein 90 | **+** | **+** |
| 28 | C0H571_PLAF7 | High molecular weight rhoptry protein 2 | **+** | **+** |
| 29 | Q8I395_PLAF7 | High molecular weight rhoptry protein 3 | **+** | **+** |
| 30 | Q8IKM6_PLAF7 | Inner membrane complex sub-compartment protein 3 | **+** | - |
| 31 | AMPL_PLAF7 | Leucine aminopeptidase | **+** | - |
| 32 | Q8I3L4_PLAF7 | Long-chain-fatty-acid--CoA ligase | **+** | - |
| 33 | C0H5L9_PLAF7 | Membrane associated histidine-rich protein 1 | **+** | - |
| 34 | Q8IDQ9_PLAF7 | Phosphoethanolamine N-methyltransferase | **+** | **+** |
| 35 | Q8IFM0_PLAF7 | Plasmodium RESA N-terminal domain-containing protein | **+** | **+** |
| 36 | Q8IKV6_PLAF7 | Rhoptry neck protein 2 | **+** | **+** |
| 37 | Q8I4R5_PLAF7 | Rhoptry neck protein 3 | **+** | - |
| 38 | C0H4V4_PLAF7 | Rhoptry neck protein 5 | **+** | - |
| 39 | Q8IBN1_PLAF7 | Rhoptry-associated leucine zipper-like protein 1 | **+** | - |
| 40 | Q8ILZ1_PLAF7 | Rhoptry-associated protein 1 | **+** | - |
| 41 | Q8I484_PLAF7 | Rhoptry-associated protein 2 | **+** | **+** |
| 42 | Q8I485_PLAF7 | Rhoptry-associated protein 3 | **+** | - |
| 43 | Q8I0U6_PLAF7 | Ring-infected erythrocyte surface antigen | **+** | **+** |
| ***Cellular signaling proteins*** | | | | |
| 44 | Q8IIV8_PLAF7 | Thioredoxin-like mero protein | **+** | - |
| 45 | Q8IIB7_PLAF7 | Ethanolamine kinase | **+** | - |
| 46 | Q8IJD0_PLAF7 | Peroxiredoxin | **+** | - |
| 47 | 1433I_PLAF7 | 14-3-3 protein I | **+** | **+** |
| 48 | Q8I328_PLAF7 | Alkaline phosphatase, putative | **+** | **-** |
| 49 | CDPK1_PLAF7 | Calcium-dependent protein kinase 1 | **+** | **-** |
| 50 | Q76NN8_PLAF7 | Calcium-transporting ATPase | **+** | **-** |
| 51 | Q7KQK0_PLAF7 | cAMP-dependent protein kinase regulatory subunit | **+** | **-** |
| 52 | Q8IIR9_PLAF7 | Casein kinase 2, alpha subunit | **+** | - |
| 53 | KC1_PLAF7 | Casein kinase I | **+** | - |
| 54 | Q7KQK6_PLAF7 | GTP-binding nuclear protein | **+** | **+** |
| 55 | Q8IJ74_PLAF7 | Haloacid dehalogenase-like hydrolase | **+** | - |
| 56 | Q7KQK2_PLAF7 | Polyubiquitin | **+** | - |
| 57 | PROF_PLAF7 | Profilin | **+** | **+** |
| 58 | Q8I2Q4_PLAF7 | Serine/threonine-protein phosphatase | **+** | - |
| 59 | Q8IL80_PLAF7 | Thioredoxin peroxidase 1 | **+** | - |
| 60 | Q8IDH5_PLAF7 | Thioredoxin-related protein, putative | **+** | **+** |
| 61 | Q8I607_PLAF7 | Ubiquitin-conjugating enzyme E2 | **+** | - |
| 62 | Q8IB31_PLAF7 | Ubiquitin-like domain-containing protein | **+** | - |
| 63 | C6KT34_PLAF7 | Cell division cycle protein 48 homologue, putative | **+** | - |
| ***Energy metabolism and protein synthesis*** | | | | |
| 64 | O96153_PLAF7 | 26S proteasome regulatory subunit RPN1, putative | **+** | **-** |
| 65 | Q8IKH3_PLAF7 | 26S proteasome regulatory subunit RPN2, putative | **+** | **-** |
| 66 | Q8II71_PLAF7 | 26S proteasome regulatory subunit RPN7, putative | **+** | **-** |
| 67 | Q8IDB0_PLAF7 | 40S ribosomal protein S15 | **+** | **+** |
| 68 | O77395_PLAF7 | 40S ribosomal protein S15A, putative | **+** | **-** |
| 69 | Q8IAX5_PLAF7 | 40S ribosomal protein S16, putative | **+** | **-** |
| 70 | Q8IIA2_PLAF7 | 40S ribosomal protein S18, putative | **+** | **-** |
| 71 | Q8IFP2_PLAF7 | 40S ribosomal protein S19 | **+** | **-** |
| 72 | O97248_PLAF7 | 40S ribosomal protein S23, putative | **+** | **+** |
| 73 | O96258_PLAF7 | 40S ribosomal protein S26 | **+** | **-** |
| 74 | Q8IKH8_PLAF7 | 40S ribosomal protein S3 | **+** | **+** |
| 75 | Q8IIU8_PLAF7 | 40S ribosomal protein S4 | **+** | **-** |
| 76 | Q8IL02_PLAF7 | 40S ribosomal protein S5 | **+** | **-** |
| 77 | Q8IBN5_PLAF7 | 40S ribosomal protein S5, putative | **+** | **-** |
| 78 | Q8IDR9_PLAF7 | 40S ribosomal protein S6 | **+** | **-** |
| 79 | Q8IET7_PLAF7 | 40S ribosomal protein S7 | **+** | **-** |
| 80 | Q8IM10_PLAF7 | 40S ribosomal protein S8 | **+** | **+** |
| 81 | Q8I3R0_PLAF7 | 40S ribosomal protein S9, putative | **+** | **-** |
| 82 | Q8II61_PLAF7 | 60S acidic ribosomal protein P0 | **+** | **-** |
| 83 | Q8ILV2_PLAF7 | 60S ribosomal protein L10, putative | **+** | **-** |
| 84 | Q8IJZ7_PLAF7 | 60S ribosomal protein L13, putative | **+** | **-** |
| 85 | Q8ILE8_PLAF7 | 60S ribosomal protein L14, putative | **+** | **-** |
| 86 | Q8IDI5_PLAF7 | 60S ribosomal protein L17, putative | **+** | **+** |
| 87 | Q8IDS6_PLAF7 | 60S ribosomal protein L18a | **+** | **-** |
| 88 | Q8I3T9_PLAF7 | 60S ribosomal protein L2 | **+** | **+** |
| 89 | Q8IE09_PLAF7 | 60S ribosomal protein L23, putative | **+** | **-** |
| 90 | C6KT23_PLAF7 | 60S ribosomal protein L27a, putative | **+** | **-** |
| 91 | Q8IJC6_PLAF7 | 60S ribosomal protein L3 | **+** | **-** |
| 92 | Q8IJK8_PLAF7 | 60S ribosomal protein L30e, putative | **+** | **+** |
| 93 | Q8IIB4_PLAF7 | 60S ribosomal protein L35ae, putative | **+** | **+** |
| 94 | Q8II62_PLAF7 | 60S ribosomal protein L38 | **+** | **-** |
| 95 | Q8I431_PLAF7 | 60S ribosomal protein L4 | **+** | **+** |
| 96 | Q8ILL3_PLAF7 | 60S ribosomal protein L5, putative | **+** | **-** |
| 97 | Q8IE85_PLAF7 | 60S ribosomal protein L6, putative | **+** | **-** |
| 98 | O97250_PLAF7 | 60S ribosomal protein L7, putative | **+** | **+** |
| 99 | Q8ILL2_PLAF7 | 60S ribosomal protein L7a | **+** | **+** |
| 100 | C6KTB4_PLAF7 | Acetyl-CoA synthetase, putative | **+** | **-** |
| 101 | AN32_PLAF7 | Acidic leucine-rich nuclear phosphoprotein 32-related protein | **+** | **-** |
| 102 | Q8I6Z1_PLAF7 | Acyl-CoA synthetase | **+** | **+** |
| 103 | SAHH_PLAF7 | Adenosylhomocysteinase | **+** | **-** |
| 104 | KAD1_PLAF7 | Adenylate kinase 1 | **+** | **-** |
| 105 | Q8IJ34_PLAF7 | ADP/ATP translocase | **+** | **-** |
| 106 | Q8IB72_PLAF7 | DnaJ protein, putative | **+** | - |
| 107 | Q8IFN9_PLAF7 | Eukaryotic initiation factor 4A-III, putative | **+** | - |
| 108 | Q8I5S6_PLAF7 | Eukaryotic translation initiation factor 3 subunit A | **+** | - |
| 109 | Q8I2X0_PLAF7 | Eukaryotic translation initiation factor 3 subunit F, putative | **+** | - |
| 110 | Q8IAZ3_PLAF7 | Eukaryotic translation initiation factor 3 subunit G | **+** | - |
| 111 | Q8IBT2_PLAF7 | Eukaryotic translation initiation factor 3 subunit I | **+** | - |
| 112 | ALF_PLAF7 | Fructose-bisphosphate aldolase | **+** | - |
| 113 | Q76NM3_PLAF7 | L-lactate dehydrogenase | **+** | **+** |
| 114 | Q8IKQ7_PLAF7 | MA3 domain-containing protein, putative | **+** | - |
| 115 | Q8I2W3_PLAF7 | Nucleosome assembly protein | **+** | - |
| 116 | OAT_PLAF7 | Ornithine aminotransferase | **+** | **+** |
| 117 | PLM1_PLAF7 | Plasmepsin I | **+** | - |
| 118 | PLM2_PLAF7 | Plasmepsin II | **+** | - |
| 119 | PLM4_PLAF7 | Plasmepsin IV | **+** | **+** |
| 120 | C0H4Y6_PLAF7 | Protein disulfide-isomerase | **+** | **+** |
| 121 | SEY1_PLAF7 | Protein SEY1 homolog | **+** | **+** |
| 122 | Q8ILY9_PLAF7 | protein-synthesizing GTPase | **+** | - |
| 123 | Q8IKF0_PLAF7 | RNA helicase | **+** | **+** |
| 124 | Q8IEK9_PLAF7 | RNA-binding protein, putative | **+** | - |
| 125 | Q7K6A4_PLAF7 | S-adenosylmethionine synthase | **+** | **+** |
| 126 | Q8ILK3_PLAF7 | 60S ribosomal protein L21 | **-** | **+** |
| 127 | O97245_PLAF7 | Glycerol-3-phosphate dehydrogenase | **-** | **+** |
| 128 | RS3A_PLAF7 | Small ribosomal subunit protein eS1 | **-** | **+** |
| 129 | Q8IAX8_PLAF7 | DNA/RNA-binding protein Alba 1 | **+** | - |
| 130 | Q8IJX8_PLAF7 | DNA/RNA-binding protein Alba 3 | **+** | - |
| 131 | Q8I546_PLAF7 | DUF4149 domain-containing protein | **+** | - |
| 132 | Q8IKK7_PLAF7 | Glyceraldehyde-3-phosphate dehydrogenase | **+** | **+** |
| 133 | Q8I0V4_PLAF7 | Endoplasmin, putative | **+** | **+** |
| 134 | Q8IEQ1_PLAF7 | 26S protease regulatory subunit 10B, putative | **+** | **-** |
| 135 | Q8IJW0_PLAF7 | 26S protease regulatory subunit 4, putative | **+** | **-** |
| 136 | Q8II60_PLAF7 | 26S protease regulatory subunit 6A, putative | **+** | **-** |
| 137 | Q8I1V1_PLAF7 | 26S protease regulatory subunit 6B, putative | **+** | **-** |
| 138 | Q8IEK3_PLAF7 | 26S protease regulatory subunit 7, putative | **+** | **+** |
| 139 | Q8I4U5_PLAF7 | 26S protease regulatory subunit 8, putative | **+** | **-** |
| ***Transport proteins*** | | | | |
| 140 | ARF1_PLAF7 | ADP-ribosylation factor 1 | **+** | **+** |
| 141 | Q8II36_PLAF7 | Aquaglyceroporin | **+** | **+** |
| 142 | Q8I5L6_PLAF7 | Clathrin heavy chain | **+** | - |
| 143 | Q8IHR4_PLAF7 | Dynamin-like protein | **+** | - |
| 144 | Q8I5M3_PLAF7 | Dynamin-like protein, putative | **+** | - |
| 145 | Q8IEI6_PLAF7 | EMP1-trafficking protein | **+** | - |
| 146 | Q8IIF0_PLAF7 | Exported protein 1 | **+** | - |
| 147 | Q8IKC8_PLAF7 | Exported protein 2 | **+** | **+** |
| 148 | Q8IKR1_PLAF7 | H(+)-exporting diphosphatase | **+** | **+** |
| 149 | Q7K6A5_PLAF7 | Multidrug resistance protein 1 | **+** | - |
| 150 | A0A5K1K8H7_PLAF7 | Ras-related protein Rab-1B | **+** | - |
| 151 | C0H516_PLAF7 | Ras-related protein RAB7 | **+** | - |
| 152 | Q8IEC8_PLAF7 | Translocation protein SEC63, putative | **+** | - |
| 153 | Q8ILA1_PLAF7 | Translocon component PTEX150 | **+** | - |
| 154 | Q8I305_PLAF7 | Major facilitator superfamily-related transporter, putative | **-** | **+** |
| 155 | Q8ILE3_PLAF7 | Voltage-dependent anion-selective channel protein, putative | **-** | **+** |
| 156 | Q8IAW0_PLAF7 | Importin subunit alpha | **+** | - |
| 157 | Q8I3M5_PLAF7 | Karyopherin beta | **+** | **+** |
| 158 | Q8I0V2_PLAF7 | ATP synthase subunit beta | **+** | **-** |
| 159 | Q9TY94_PLAF7 | ATP-dependent RNA helicase UAP56 | **+** | **+** |
| 160 | Q8I0P6_PLAF7 | Elongation factor 1-alpha | **+** | **+** |
| 161 | Q8I320_PLAF7 | Elongation factor 1-beta | **+** | - |
| 162 | O97319_PLAF7 | Elongation factor 1-delta, putative | **+** | - |
| 163 | A0A5K1K967_PLAF7 | Elongation factor 1-gamma, putative | **+** | - |
| 164 | Q8IKW5_PLAF7 | Elongation factor 2 | **+** | - |
| 165 | Q8IC43_PLAF7 | Small GTP-binding protein sar1 | **+** | - |
| 166 | Q8I487_PLAF7 | Skeleton-binding protein 1 | **+** | - |

**Supplementary** **table 3.** Protein identified in conglomerate 1 of erythrocytes with the schizont forms of *P. falciparum FCB-2.*

| ***Conglomerate 1*** **S*chizont*** | | | | |
| --- | --- | --- | --- | --- |
| ***N°*** | ***ID*** | ***Proteína*** | ***Condición*** | |
|  |  |  | **Control** | ***d-G6PD*** |
| ***Proteins involved in DNA and RNA processing*** | | | | |
| 1 | PGK_PLAF7 | Phosphoglycerate kinase | **+** | **-** |
| 2 | Q8IE67_PLAF7 | Phosphoribosylpyrophosphate synthetase | **+** | **-** |
| 3 | Q8I2Z8_PLAF7 | Probable ATP-dependent 6-phosphofructokinase | **+** | **-** |
| 4 | Q8IJS1_PLAF7 | Hypoxanthine phosphoribosyltransferase | **-** | **+** |
| 5 | A0A5K1K910_PLAF7 | aspartate carbamoyltransferase | **+** | **-** |
| 6 | Q8IIV1_PLAF7 | Histone H2B | **+** | **+** |
| 7 | Q8IIV2_PLAF7 | Histone H4 | **+** | **+** |
| 8 | TBB_PLAF7 | Tubulin beta chain | **+** | **-** |
| ***Adhesion proteins*** | | | | |
| 9 | O77310_PLAF7 | Cytoadherence linked asexual protein 3.1 | **+** | **-** |
| 10 | Q8I2G2_PLAF7 | Cytoadherence linked asexual protein 9 | **+** | **-** |
| ***Effector proteins*** | | | | |
| 11 | ACT1_PLAF7 | Actin-1 | **+** | **+** |
| 12 | ENO_PLAF7 | Enolase | **+** | **-** |
| 13 | Q8IEU2_PLAF7 | Gamete antigen 27/25 | **+** | **-** |
| 14 | Q8IIJ8_PLAF7 | Heat shock protein 101 | **+** | **-** |
| 15 | Q8IJN9_PLAF7 | Heat shock protein 60 | **+** | **-** |
| 16 | Q8II24_PLAF7 | Heat shock protein 70 | **+** | **+** |
| 17 | Q8IC05_PLAF7 | Heat shock protein 90 | **+** | **-** |
| 18 | C0H571_PLAF7 | High molecular weight rhoptry protein 2 | **+** | **+** |
| 19 | Q8I395_PLAF7 | High molecular weight rhoptry protein 3 | **+** | **+** |
| 20 | Q8I3L4_PLAF7 | Long-chain-fatty-acid--CoA ligase | **+** | **-** |
| 21 | Q8IFM0_PLAF7 | Plasmodium RESA N-terminal domain-containing protein | **+** | **-** |
| 22 | Q8I4R5_PLAF7 | Rhoptry neck protein 3 | **+** | **-** |
| 23 | Q8ILZ1_PLAF7 | Rhoptry-associated protein 1 | **+** | **+** |
| 24 | Q8I484_PLAF7 | Rhoptry-associated protein 2 | **+** | **+** |
| 25 | Q8I485_PLAF7 | Rhoptry-associated protein 3 | **+** | **+** |
| ***Cellular signaling proteins*** | | | | |
| 26 | 1433I_PLAF7 | 14-3-3 protein I | **+** | **-** |
| 27 | Q8IIR9_PLAF7 | Casein kinase 2, alpha subunit | **+** | **-** |
| 28 | Q7KQK6_PLAF7 | GTP-binding nuclear protein | **+** | **-** |
| 29 | Q7KQK2_PLAF7 | Polyubiquitin | **+** | **+** |
| 30 | Q8IDH5_PLAF7 | Thioredoxin-related protein, putative | **+** | **+** |
| 31 | Q8IB31_PLAF7 | Ubiquitin-like domain-containing protein | **+** | **-** |
| 32 | Q8IKJ1_PLAF7 | Serine/threonine protein phosphatase UIS2, putative | **-** | **+** |
| 33 | THIO2_PLAF7 | Thioredoxin 2 | **-** | **+** |
| 34 | Q8IL80_PLAF7 | Thioredoxin peroxidase 1 | **-** | **+** |
| 35 | Q8IBA0_PLAF7 | Receptor for activated c kinase | **+** | **-** |
| ***Energy metabolism and protein synthesis*** | | | | |
| 36 | Q8IDB0_PLAF7 | 40S ribosomal protein S15 | **+** | **-** |
| 37 | Q8IAX5_PLAF7 | 40S ribosomal protein S16, putative | **+** | **-** |
| 38 | Q8IFP2_PLAF7 | 40S ribosomal protein S19 | **+** | **-** |
| 39 | O97248_PLAF7 | 40S ribosomal protein S23, putative | **+** | **-** |
| 40 | Q8IKH8_PLAF7 | 40S ribosomal protein S3 | **+** | **-** |
| 41 | Q8IL02_PLAF7 | 40S ribosomal protein S5 | **+** | **-** |
| 42 | Q8IM10_PLAF7 | 40S ribosomal protein S8 | **+** | **-** |
| 43 | Q8ILV2_PLAF7 | 60S ribosomal protein L10, putative | **+** | **+** |
| 44 | Q8ILE8_PLAF7 | 60S ribosomal protein L13 | **+** | **-** |
| 45 | Q8IDI5_PLAF7 | 60S ribosomal protein L14, putative | **+** | **-** |
| 46 | Q8IDI5_PLAF7 | 60S ribosomal protein L17, putative | **+** | **-** |
| 47 | C0H5G3_PLAF7 | 60S ribosomal protein L18-2, putative | **+** | **-** |
| 48 | Q8I3T9_PLAF7 | 60S ribosomal protein L2 | **+** | **-** |
| 49 | Q8ILK3_PLAF7 | 60S ribosomal protein L21 | **+** | **-** |
| 50 | Q8IE09_PLAF7 | 60S ribosomal protein L23, putative | **+** | **-** |
| 51 | Q8IJC6_PLAF7 | 60S ribosomal protein L3 | **+** | **+** |
| 52 | Q8I431_PLAF7 | 60S ribosomal protein L4 | **+** | **-** |
| 53 | Q8ILL3_PLAF7 | 60S ribosomal protein L5, putative | **+** | **-** |
| 54 | Q8IE85_PLAF7 | 60S ribosomal protein L6, putative | **+** | **-** |
| 55 | O97250_PLAF7 | 60S ribosomal protein L7, putative | **+** | **+** |
| 56 | Q8ILL2_PLAF7 | 60S ribosomal protein L7a | **+** | **-** |
| 57 | Q8I6Z1_PLAF7 | Acyl-CoA synthetase | **+** | **-** |
| 58 | KAD1_PLAF7 | Adenylate kinase 1 | **+** | **-** |
| 59 | Q8IJ34_PLAF7 | ADP/ATP translocase | **+** | **-** |
| 60 | ARF1_PLAF7 | ADP-ribosylation factor 1 | **+** | **+** |
| 61 | Q8IBD0_PLAF7 | CRA domain-containing protein, putative | **+** | **-** |
| 62 | ALF_PLAF7 | Fructose-bisphosphate aldolase | **+** | **-** |
| 63 | Q8ILT0_PLAF7 | Glutamate dehydrogenase | **+** | **-** |
| 64 | Q76NM3_PLAF7 | L-lactate dehydrogenase | **+** | **+** |
| 65 | Q8I2W3_PLAF7 | Nucleosome assembly protein | **+** | **-** |
| 66 | OAT_PLAF7 | Ornithine aminotransferase | **+** | **-** |
| 67 | Q8IDR1_PLAF7 | phosphoenolpyruvate carboxykinase (ATP) | **+** | **-** |
| 68 | Q8IDQ9_PLAF7 | Phosphoethanolamine N-methyltransferase | **+** | **-** |
| 69 | PLM1_PLAF7 | Plasmepsin I | **+** | **+** |
| 70 | PLM4_PLAF7 | Plasmepsin IV | **+** | **+** |
| 71 | C0H4A6_PLAF7 | Ribosomal protein L15 | **+** | **-** |
| 72 | Q8IKF0_PLAF7 | RNA helicase | **+** | **-** |
| 73 | Q7K6A4_PLAF7 | S-adenosylmethionine synthase | **+** | **-** |
| 74 | RS3A_PLAF7 | Small ribosomal subunit protein eS1 | **+** | **-** |
| 75 | Q8IIB4_PLAF7 | 60S ribosomal protein L35ae, putative | **-** | **+** |
| 76 | PLM3_PLAF7 | Plasmepsin III | **-** | **+** |
| 77 | Q8IAX8_PLAF7 | DNA/RNA-binding protein Alba 1 | **+** | **-** |
| 78 | Q8IJX8_PLAF7 | DNA/RNA-binding protein Alba 3 | **+** | **-** |
| 79 | Q8I546_PLAF7 | DUF4149 domain-containing protein | **+** | **+** |
| 80 | Q8IKK7_PLAF7 | Glyceraldehyde-3-phosphate dehydrogenase | **+** | **-** |
| 81 | Q8I0V4_PLAF7 | Endoplasmin, putative | **+** | **-** |
| 82 | Q8IJW0_PLAF7 | 26S protease regulatory subunit 4, putative | **+** | **-** |
| 83 | Q8I1V1_PLAF7 | 26S protease regulatory subunit 6B, putative | **+** | **-** |
| 84 | Q8IEK3_PLAF7 | 26S protease regulatory subunit 7, putative | **+** | **-** |
| ***Transport proteins*** | | | | |
| 85 | Q8II36_PLAF7 | Aquaglyceroporin | **+** | **-** |
| 86 | Q8IEI6_PLAF7 | EMP1-trafficking protein | **+** | **-** |
| 87 | Q8IIF0_PLAF7 | Exported protein 1 | **+** | **-** |
| 88 | Q8IKC8_PLAF7 | Exported protein 2 | **+** | **+** |
| 89 | Q7K6A5_PLAF7 | Multidrug resistance protein 1 | **+** | **+** |
| 90 | A0A5K1K8H7_PLAF7 | Ras-related protein Rab-1B | **+** | **-** |
| 91 | Q8IIU7_PLAF7 | Translocon component PTEX88 | **+** | **-** |
| 92 | Q8ILE3_PLAF7 | Voltage-dependent anion-selective channel protein, putative | **+** | **+** |
| 93 | Q8IAQ8_PLAF7 | V-type proton ATPase subunit a, putative | **+** | **-** |
| 94 | Q8I305_PLAF7 | Major facilitator superfamily-related transporter, putative | **-** | **+** |
| 95 | Q8I3M5_PLAF7 | Karyopherin beta | **-** | **+** |
| 96 | Q8I0P6_PLAF7 | Elongation factor 1-alpha | **+** | **-** |
| 97 | Q8I0V2_PLAF7 | ATP synthase subunit beta | **+** | **+** |
| 98 | Q9TY94_PLAF7 | ATP-dependent RNA helicase UAP56 | **+** | **-** |

**Supplementary** **table 4.** Protein identified in conglomerate 2 of erythrocytes with the ring forms of *P. falciparum FCB-2.*

| ***Conglomerate 2 Ring*** | | | | |
| --- | --- | --- | --- | --- |
| ***N°*** | ***ID*** | ***Protein name*** | ***Condition*** | |
|  |  |  | ***Control*** | ***d-G6PD*** |
| ***Proteins involved in DNA and RNA processing*** | | | | |
| 1 | Q8IIW0_PLAF7 | Chromatin remodeling protein | **+** | **-** |
| 2 | Q8IBA0_PLAF7 | Receptor for activated c kinase | **+** | **-** |
| 3 | Q8IIQ1_PLAF7 | Replication factor C subunit 5, putative | **+** | **-** |
| 4 | Q8IIU3_PLAF7 | RuvB-like helicase | **+** | **-** |
| 5 | Q8I3Q6_PLAF7 | Single-stranded DNA-binding protein, putative | **+** | **-** |
| 6 | C6KT82_PLAF7 | SNF2 helicase, putative | **+** | **-** |
| 7 | Q8II73_PLAF7 | Spermidine synthase | **+** | **-** |
| 8 | C6KSV9_PLAF7 | SWIB/MDM2 domain-containing protein | **+** | **-** |
| 9 | Q8ILC8_PLAF7 | DNA topoisomerase 2 | **+** | **-** |
| 10 | Q8IB67_PLAF7 | Histone acetyltransferase GCN5 | **+** | **-** |
| 11 | Q8IIV1_PLAF7 | Histone H2B | **+** | **-** |
| 12 | Q8IIV2_PLAF7 | Histone H4 | **+** | **+** |
| 13 | Q8IJS1_PLAF7 | Hypoxanthine phosphoribosyltransferase | **+** | **-** |
| 14 | Q8I6T2_PLAF7 | Isocitrate dehydrogenase [NADP] | **+** | **-** |
| 15 | Q8ILJ7_PLAF7 | Phosphoenolpyruvate carboxylase | **+** | **-** |
| 16 | Q8IDQ9_PLAF7 | Phosphoethanolamine N-methyltransferase | **+** | **-** |
| 17 | Q8IE67_PLAF7 | Phosphoribosylpyrophosphate synthetase | **+** | **-** |
| 18 | PLM1_PLAF7 | Plasmepsin I | **+** | **+** |
| 19 | Q8IJS7_PLAF7 | PRE-binding protein | **+** | **-** |
| 20 | Q8I2Z8_PLAF7 | Probable ATP-dependent 6-phosphofructokinase | **+** | **-** |
| 21 | Q6ZLZ9_PLAF7 | Tubulin alpha chain | **+** | **-** |
| 22 | TBB_PLAF7 | Tubulin beta chain | **+** | **-** |
| ***Adhesion proteins*** | | | | |
| 23 | Q7KQK5_PLAF7 | Apical membrane antigen 1 | **+** | **-** |
| 24 | O77310_PLAF7 | Cytoadherence linked asexual protein 3.1 | **+** | **+** |
| 25 | O77309_PLAF7 | Cytoadherence linked asexual protein 3.2 | **+** | **-** |
| 26 | Q8I2G2_PLAF7 | Cytoadherence linked asexual protein 9 | **+** | **+** |
| 27 | Q76NM5_PLAF7 | Erythrocyte binding antigen-140 | **+** | **-** |
| 28 | Q8IBE8_PLAF7 | Erythrocyte binding antigen-175 | **+** | **-** |
| 29 | Q8I2B4_PLAF7 | Erythrocyte binding antigen-181 | **+** | **-** |
| 30 | Q8I0U8_PLAF7 | Merozoite surface protein 1 | **+** | **-** |
| 31 | MSP9_PLAF7 | Merozoite surface protein 9 | **+** | **+** |
| 32 | PF92_PLAF7 | Merozoite surface protein P92 | **+** | **-** |
| 33 | RH1_PLAF7 | Reticulocyte-binding protein homolog 1 | **+** | **-** |
| 34 | P113_PLAF7 | Surface protein P113 | **+** | **-** |
| ***Effector proteins*** | | | | |
| 35 | ACT1_PLAF7 | Actin-1 | **+** | **-** |
| 36 | ENO_PLAF7 | Enolase | **+** | **-** |
| 37 | Q8IEU2_PLAF7 | Gamete antigen 27/25 | **+** | **-** |
| 38 | A0A5K1K8J3_PLAF7 | Glideosome-associated connector | **+** | **-** |
| 39 | Q8IIJ8_PLAF7 | Heat shock protein 101 | **+** | **-** |
| 40 | Q8IJN9_PLAF7 | Heat shock protein 60 | **+** | **-** |
| 41 | Q8II24_PLAF7 | Heat shock protein 70 | **+** | **-** |
| 42 | Q8IC05_PLAF7 | Heat shock protein 90 | **+** | **-** |
| 43 | Q8III6_PLAF7 | Heat shock protein 90, putative | **+** | **-** |
| 44 | C0H571_PLAF7 | High molecular weight rhoptry protein 2 | **+** | **+** |
| 45 | Q8I395_PLAF7 | High molecular weight rhoptry protein 3 | **+** | **+** |
| 46 | Q8IL88_PLAF7 | HSP40, subfamily A | **+** | **-** |
| 47 | Q8IKF6_PLAF7 | Kelch domain-containing protein, putative | **+** | **-** |
| 48 | O96275_PLAF7 | Liver stage antigen 3 | **+** | **-** |
| 49 | Q8I3L4_PLAF7 | Long-chain-fatty-acid--CoA ligase | **+** | **-** |
| 50 | C0H5L9_PLAF7 | Membrane associated histidine-rich protein 1 | **+** | **-** |
| 51 | Q8IFM0_PLAF7 | Plasmodium RESA N-terminal domain-containing protein | **+** | **-** |
| 52 | Q8IKV6_PLAF7 | Rhoptry neck protein 2 | **+** | **+** |
| 53 | Q8I4R5_PLAF7 | Rhoptry neck protein 3 | **+** | **-** |
| 54 | Q8IIK5_PLAF7 | Rhoptry neck protein 4 | **+** | **-** |
| 55 | C0H4V4_PLAF7 | Rhoptry neck protein 5 | **+** | **-** |
| 56 | Q8IBN1_PLAF7 | Rhoptry-associated leucine zipper-like protein 1 | **+** | **+** |
| 57 | Q8ILZ1_PLAF7 | Rhoptry-associated protein 1 | **+** | **+** |
| 58 | Q8I484_PLAF7 | Rhoptry-associated protein 2 | **+** | **+** |
| 59 | Q8I485_PLAF7 | Rhoptry-associated protein 3 | **+** | **+** |
| 60 | Q8I2G1_PLAF7 | Ring-exported protein 1 | **+** | **-** |
| 61 | Q8I0U6_PLAF7 | Ring-infected erythrocyte surface antigen | **+** | **-** |
| 62 | Q8IIV8_PLAF7 | Thioredoxin-like mero protein | **+** | **-** |
| ***Cellular signaling proteins*** | | | | |
| 63 | Q8IKJ1_PLAF7 | Serine/threonine protein phosphatase UIS2, putative | **+** | **-** |
| 64 | Q8IKE5_PLAF7 | Shewanella-like protein phosphatase 1, putative | **+** | **-** |
| 65 | THIO2_PLAF7 | Thioredoxin 2 | **+** | **-** |
| 66 | Q8IL80_PLAF7 | Thioredoxin peroxidase 1 | **+** | **+** |
| 67 | Q8IDH5_PLAF7 | Thioredoxin-related protein, putative | **+** | **+** |
| 68 | Q8IJD0_PLAF7 | Peroxiredoxin | **+** | **-** |
| 69 | 1433I_PLAF7 | 14-3-3 protein I | **+** | **-** |
| 70 | DNPEP_PLAF7 | Aspartyl aminopeptidase | **+** | **-** |
| 71 | CDPK1_PLAF7 | Calcium-dependent protein kinase 1 | **+** | **+** |
| 72 | Q7KQK0_PLAF7 | cAMP-dependent protein kinase regulatory subunit | **+** | **-** |
| 73 | Q8IIR9_PLAF7 | Casein kinase 2, alpha subunit | **+** | **-** |
| 74 | Q7KQK6_PLAF7 | GTP-binding nuclear protein | **+** | **-** |
| 75 | Q8IB31_PLAF7 | Ubiquitin-like domain-containing protein | **+** | **-** |
| 76 | Q7KQK2_PLAF7 | Polyubiquitin | **-** | **+** |
| 77 | C6KT34_PLAF7 | Cell division cycle protein 48 homologue, putative | **+** | **-** |
| 78 | Q8II87_PLAF7 | CRAL/TRIO domain-containing protein, putative | **+** | **-** |
| ***Energy metabolism and protein synthesis*** | | | | |
| 79 | Q8IJM0_PLAF7 | 26S proteasome regulatory subunit p55, putative | **+** | **-** |
| 80 | O96153_PLAF7 | 26S proteasome regulatory subunit RPN1, putative | **+** | **+** |
| 81 | Q8ID28_PLAF7 | 26S proteasome regulatory subunit RPN11, putative | **+** | **+** |
| 82 | O77345_PLAF7 | 26S proteasome regulatory subunit RPN12, putative | **+** | **-** |
| 83 | Q8IKH3_PLAF7 | 26S proteasome regulatory subunit RPN2, putative | **+** | **-** |
| 84 | Q8IDB0_PLAF7 | 40S ribosomal protein S15 | **+** | **-** |
| 85 | Q8IAX5_PLAF7 | 40S ribosomal protein S16, putative | **+** | **-** |
| 86 | Q8I502_PLAF7 | 40S ribosomal protein S17, putative | **+** | **-** |
| 87 | Q8IIA2_PLAF7 | 40S ribosomal protein S18, putative | **+** | **-** |
| 88 | Q8IFP2_PLAF7 | 40S ribosomal protein S19 | **+** | **-** |
| 89 | O96258_PLAF7 | 40S ribosomal protein S26 | **+** | **-** |
| 90 | Q8IKH8_PLAF7 | 40S ribosomal protein S3 | **+** | **-** |
| 91 | Q8IL02_PLAF7 | 40S ribosomal protein S5 | **+** | **-** |
| 92 | Q8IBN5_PLAF7 | 40S ribosomal protein S5, putative | **+** | **-** |
| 93 | Q8IDR9_PLAF7 | 40S ribosomal protein S6 | **+** | **-** |
| 94 | Q8IM10_PLAF7 | 40S ribosomal protein S8 | **+** | **-** |
| 95 | Q8I3R0_PLAF7 | 40S ribosomal protein S9, putative | **+** | **-** |
| 96 | Q8I0V3_PLAF7 | 60 kDa chaperonin | **+** | **+** |
| 97 | Q8II61_PLAF7 | 60S acidic ribosomal protein P0 | **+** | **-** |
| 98 | Q8IIX0_PLAF7 | 60S acidic ribosomal protein P1, putative | **+** | **-** |
| 99 | Q8IJZ7_PLAF7 | 60S ribosomal protein L13, putative | **+** | **-** |
| 100 | Q8ILE8_PLAF7 | 60S ribosomal protein L14, putative | **+** | **-** |
| 101 | Q8IDI5_PLAF7 | 60S ribosomal protein L17, putative | **+** | **-** |
| 102 | C0H5G3_PLAF7 | 60S ribosomal protein L18-2, putative | **+** | **-** |
| 103 | Q8IDS6_PLAF7 | 60S ribosomal protein L18a | **+** | **-** |
| 104 | C6KSY6_PLAF7 | 60S ribosomal protein L19 | **+** | **-** |
| 105 | Q8I3T9_PLAF7 | 60S ribosomal protein L2 | **+** | **-** |
| 106 | Q8ILK3_PLAF7 | 60S ribosomal protein L21 | **+** | **-** |
| 107 | Q8IE09_PLAF7 | 60S ribosomal protein L23, putative | **+** | **-** |
| 108 | C6KT23_PLAF7 | 60S ribosomal protein L27a, putative | **+** | **-** |
| 109 | Q8IJC6_PLAF7 | 60S ribosomal protein L3 | **+** | **-** |
| 110 | Q8I431_PLAF7 | 60S ribosomal protein L4 | **+** | **-** |
| 111 | Q8ILL3_PLAF7 | 60S ribosomal protein L5, putative | **+** | **-** |
| 112 | Q8IE85_PLAF7 | 60S ribosomal protein L6, putative | **+** | **-** |
| 113 | O97250_PLAF7 | 60S ribosomal protein L7, putative | **+** | **-** |
| 114 | Q8ILL2_PLAF7 | 60S ribosomal protein L7a | **+** | **+** |
| 115 | C6KTB4_PLAF7 | Acetyl-CoA synthetase, putative | **+** | **-** |
| 116 | Q8I6Z1_PLAF7 | Acyl-CoA synthetase | **+** | **-** |
| 117 | KAD1_PLAF7 | Adenylate kinase 1 | **+** | **-** |
| 118 | AMPN_PLAF7 | Aminopeptidase N | **+** | **-** |
| 119 | Q8IIR6_PLAF7 | DnaJ homolog subfamily C member 16 | **+** | **-** |
| 120 | Q8IB72_PLAF7 | DnaJ protein, putative | **+** | **-** |
| 121 | Q8IE22_PLAF7 | ER lumen protein retaining receptor 1, putative | **+** | **-** |
| 122 | C0H5B1_PLAF7 | Eukaryotic translation initation factor 4 gamma | **+** | **-** |
| 123 | Q8I5S6_PLAF7 | Eukaryotic translation initiation factor 3 subunit A | **+** | **-** |
| 124 | Q8I3T1_PLAF7 | Eukaryotic translation initiation factor 3 subunit B | **+** | **-** |
| 125 | Q8IJW4_PLAF7 | Eukaryotic translation initiation factor 3 subunit D | **+** | **-** |
| 126 | Q8I1Q5_PLAF7 | Eukaryotic translation initiation factor 3 subunit M | **+** | **-** |
| 127 | Q8I3T4_PLAF7 | FACT complex subunit | **+** | **-** |
| 128 | Q8IL56_PLAF7 | FACT complex subunit SSRP1 | **+** | **-** |
| 129 | ALF_PLAF7 | Fructose-bisphosphate aldolase | **+** | **-** |
| 130 | Q8ILT0_PLAF7 | Glutamate dehydrogenase | **+** | **-** |
| 131 | C0H551_PLAF7 | Glutamine synthetase, putative | **+** | **-** |
| 132 | GSHR_PLAF7 | Glutathione reductase | **+** | **-** |
| 133 | O97245_PLAF7 | Glycerol-3-phosphate dehydrogenase | **+** | **-** |
| 134 | RL44_PLAF7 | Large ribosomal subunit protein P2 | **+** | **-** |
| 135 | AMPL_PLAF7 | Leucine aminopeptidase | **+** | **-** |
| 136 | Q76NM3_PLAF7 | L-lactate dehydrogenase | **+** | **+** |
| 137 | Q8III3_PLAF7 | Nucleolar protein 56, putative | **+** | **-** |
| 138 | Q8I2W3_PLAF7 | Nucleosome assembly protein | **+** | **+** |
| 139 | OAT_PLAF7 | Ornithine aminotransferase | **+** | **-** |
| 140 | PLM3_PLAF7 | Plasmepsin III | **+** | **-** |
| 141 | PLM4_PLAF7 | Plasmepsin IV | **+** | **-** |
| 142 | Q8I1X5_PLAF7 | Pre-mRNA-processing-splicing factor 8, putative | **+** | **-** |
| 143 | Q8I374_PLAF7 | Proteasome activator 28 subunit beta, putative | **+** | **-** |
| 144 | Q8IDG2_PLAF7 | Proteasome subunit alpha type | **+** | **-** |
| 145 | Q8IK90_PLAF7 | Proteasome subunit alpha type-1, putative | **+** | **-** |
| 146 | C6KST3_PLAF7 | Proteasome subunit alpha type-2, putative | **+** | **-** |
| 147 | O77396_PLAF7 | Proteasome subunit alpha type-3, putative | **+** | **-** |
| 148 | Q8IAR3_PLAF7 | Proteasome subunit alpha type-6, putative | **+** | **-** |
| 149 | Q8IJT1_PLAF7 | Proteasome subunit beta | **+** | **-** |
| 150 | Q8I0U7_PLAF7 | Proteasome subunit beta type-6, putative | **+** | **-** |
| 151 | C0H4Y6_PLAF7 | Protein disulfide isomerase | **+** | **-** |
| 152 | C0H4A6_PLAF7 | Ribosomal protein L15 | **+** | **-** |
| 153 | Q8IKF0_PLAF7 | RNA helicase | **+** | **+** |
| 154 | Q8II69_PLAF7 | rRNA (Cytosine-C(5))-methyltransferase, putative | **+** | **-** |
| 155 | Q7K6A4_PLAF7 | S-adenosylmethionine synthase | **+** | **-** |
| 156 | RS3A_PLAF7 | Small ribosomal subunit protein eS1 | **+** | **-** |
| 157 | Q8II43_PLAF7 | T-complex protein 1 subunit alpha | **+** | **-** |
| 158 | C0H5I7_PLAF7 | T-complex protein 1 subunit delta | **+** | **-** |
| 159 | C0H5I7_PLAF7 | T-complex protein 1 subunit eta | **+** | **-** |
| 160 | O96220_PLAF7 | T-complex protein 1 subunit theta | **+** | **-** |
| 161 | C6KST5_PLAF7 | T-complex protein 1 subunit zeta | **+** | **-** |
| 162 | Q8IJP9_PLAF7 | Transcriptional coactivator ADA2 | **+** | **-** |
| 163 | ASNA_PLAF7 | ATPase ASNA1 homolog | **+** | **-** |
| 164 | Q8I3P6_PLAF7 | ATP-dependent RNA helicase | **+** | **-** |
| 165 | Q8IL13_PLAF7 | ATP-dependent RNA helicase DDX5, putative | **+** | **-** |
| 166 | Q8I3B4_PLAF7 | ATP-dependent RNA helicase DDX60, putative | **+** | **-** |
| 167 | Q9TY94_PLAF7 | ATP-dependent RNA helicase UAP56 | **+** | **-** |
| 168 | Q8IAX8_PLAF7 | DNA/RNA-binding protein Alba 1 | **+** | **-** |
| 169 | Q8IJX8_PLAF7 | DNA/RNA-binding protein Alba 3 | **+** | **-** |
| 170 | A0A5K1K8Y8_PLAF7 | DNA/RNA-binding protein Alba 4 | **+** | **-** |
| 171 | Q8I5X9_PLAF7 | DNA-directed RNA polymerase subunit beta | **+** | **-** |
| 172 | Q8I546_PLAF7 | DUF4149 domain-containing protein | **+** | **+** |
| 173 | Q8IKK7_PLAF7 | Glyceraldehyde-3-phosphate dehydrogenase | **+** | **-** |
| 174 | Q8I0V4_PLAF7 | Endoplasmin, putative | **+** | **+** |
| 175 | Q8IEQ1_PLAF7 | 26S protease regulatory subunit 10B, putative | **+** | **-** |
| 176 | Q8IJW0_PLAF7 | 26S protease regulatory subunit 4, putative | **+** | **-** |
| 177 | Q8II60_PLAF7 | 26S protease regulatory subunit 6A, putative | **+** | **-** |
| 178 | Q8I1V1_PLAF7 | 26S protease regulatory subunit 6B, putative | **+** | **+** |
| ***Transport proteins*** | | | | |
| 179 | Q8I6Z4_PLAF7 | ABC transporter E family member 1, putative | **+** | **-** |
| 180 | ARF1_PLAF7 | ADP-ribosylation factor 1 | **+** | **-** |
| 181 | Q8II36_PLAF7 | Aquaglyceroporin | **+** | **+** |
| 182 | C6KSR5_PLAF7 | Coatomer alpha subunit, putative | **+** | **-** |
| 183 | Q8IHR4_PLAF7 | Dynamin-like protein | **+** | **-** |
| 184 | Q8I5M3_PLAF7 | Dynamin-like protein, putative | **+** | **-** |
| 185 | Q8I3M5_PLAF7 | Karyopherin beta | **+** | **-** |
| 186 | Q8I305_PLAF7 | Major facilitator superfamily-related transporter, putative | **+** | **-** |
| 187 | Q7K6A5_PLAF7 | Multidrug resistance protein 1 | **+** | **-** |
| 188 | Q8IKZ6_PLAF7 | Multidrug resistance protein 2 | **+** | **-** |
| 189 | A0A143ZZK9_PLAF7 | Non-SERCA-type Ca2+ -transporting P-ATPase | **+** | **-** |
| 190 | Q8I2Q0_PLAF7 | Parasitophorous vacuolar protein 5, putative | **+** | **-** |
| 191 | Q76NM4_PLAF7 | Ras-related protein Rab-11A | **+** | **-** |
| 192 | Q8I5A9_PLAF7 | Ras-related protein Rab-2 | **+** | **-** |
| 193 | A0A143ZWB4_PLAF7 | SNARE protein, putative | **+** | **-** |
| 194 | Q8ILA1_PLAF7 | Translocon component PTEX150 | **+** | **-** |
| 195 | Q8IIU7_PLAF7 | Translocon component PTEX88 | **+** | **-** |
| 196 | O77313_PLAF7 | Vesicle-fusing ATPase | **+** | **-** |
| 197 | VATA_PLAF7 | V-type proton ATPase catalytic subunit A | **+** | **-** |
| 198 | Q8IKJ0_PLAF7 | V-type proton ATPase subunit | **+** | **-** |
| 199 | Q8IAQ8_PLAF7 | V-type proton ATPase subunit a, putative | **+** | **-** |
| 200 | A0A5K1K8H7_PLAF7 | Ras-related protein Rab-1B | **-** | **+** |
| 201 | Q8IIF0_PLAF7 | Exported protein 1 | **+** | **+** |
| 202 | Q8IKC8_PLAF7 | Exported protein 2 | **+** | **+** |
| 203 | Q8IKR1_PLAF7 | H(+)-exporting diphosphatase | **+** | **-** |
| 204 | Q8I298_PLAF7 | StAR-related lipid transfer protein | **+** | **-** |
| 205 | Q8IJ76_PLAF7 | Early transcribed membrane protein 10.2 | **+** | **-** |
| 206 | A0A5K1K7X4_PLAF7 | Early transcribed membrane protein 5 | **+** | **-** |
| 207 | Q8I0P6_PLAF7 | Elongation factor 1-alpha | **+** | **+** |
| 208 | O97319_PLAF7 | Elongation factor 1-delta, putative | **+** | **-** |
| 209 | A0A5K1K967_PLAF7 | Elongation factor 1-gamma, putative | **+** | **-** |
| 210 | Q8I0V2_PLAF7 | ATP synthase subunit beta | **-** | **+** |
| 211 | O96252_PLAF7 | ATP synthase subunit alpha | **+** | **-** |

**Supplementary** **table 5.** Protein identified in conglomerate 2 of erythrocytes with the trophozoite forms of *P. falciparum FCB-2.*

| ***Conglomerate 2 Trophozoite*** | | | | |
| --- | --- | --- | --- | --- |
| ***N°*** | ***ID*** | ***Protein name*** | ***Condition*** | |
|  |  |  | ***Control*** | ***d-G6PD*** |
| ***Proteins involved in DNA and RNA processing*** | | | | |
| 1 | Q8IIV2_PLAF7 | Histone H4 | **+** | **+** |
| 2 | Q8IIV1_PLAF7 | Histone H2B | **+** | **-** |
| 3 | Q8IE67_PLAF7 | Phosphoribosylpyrophosphate synthetase | **+** | **-** |
| 4 | O77325_PLAF7 | Pre-mRNA-processing factor 19 | **+** | **-** |
| 5 | C6KTA4_PLAF7 | Pyruvate kinase | **+** | **-** |
| 6 | Q8IKF0_PLAF7 | RNA helicase | **+** | **-** |
| 7 | Q8II73_PLAF7 | Spermidine synthase | **+** | **-** |
| 8 | Q8I2Z8_PLAF7 | Probable ATP-dependent 6-phosphofructokinase | **+** | **+** |
| ***Adhesion proteins*** | | | | |
| 9 | O77309_PLAF7 | Cytoadherence linked asexual protein 3.2 | **+** | **-** |
| 10 | Q8I2G2_PLAF7 | Cytoadherence linked asexual protein 9 | **+** | **-** |
| 11 | Q8I0U8_PLAF7 | Merozoite surface protein 1 | **+** | **-** |
| 12 | PF92_PLAF7 | Merozoite surface protein P92 | **+** | **-** |
| 13 | C6KSR6_PLAF7 | VFT protein | **+** | **-** |
| 14 | O77310_PLAF7 | Cytoadherence linked asexual protein 3.1 | **+** | **+** |
| 15 | MSP9_PLAF7 | Merozoite surface protein 9 | **+** | **+** |
| 16 | P113_PLAF7 | Surface protein P113 | **+** | **+** |
| ***Effector proteins*** | | | | |
| 17 | Q8IIJ8_PLAF7 | Heat shock protein 101 | **+** | **+** |
| 18 | Q8II24_PLAF7 | Heat shock protein 70 | **+** | **+** |
| 19 | C0H571_PLAF7 | High molecular weight rhoptry protein 2 | **+** | **+** |
| 20 | Q8I395_PLAF7 | High molecular weight rhoptry protein 3 | **+** | **+** |
| 21 | Q8IDQ9_PLAF7 | Phosphoethanolamine N-methyltransferase | **+** | **-** |
| 22 | Q8I4R5_PLAF7 | Rhoptry neck protein 3 | **+** | **+** |
| 23 | Q8ILZ1_PLAF7 | Rhoptry-associated protein 1 | **+** | **+** |
| 24 | Q8I484_PLAF7 | Rhoptry-associated protein 2 | **+** | **+** |
| 25 | Q8I485_PLAF7 | Rhoptry-associated protein 3 | **+** | **+** |
| 26 | CADF1_PLAF7 | Cofilin/actin-depolymerizing factor homolog 1 | **+** | **-** |
| 27 | Q8IEI6_PLAF7 | EMP1-trafficking protein | **+** | **-** |
| 28 | A0A5K1K8J3_PLAF7 | Glideosome-associated protein 50 | **+** | **-** |
| 29 | Q8IKS4_PLAF7 | Hemolysin III | **+** | **-** |
| 30 | Q8IIK1_PLAF7 | Phospholipid-transporting ATPase, putative | **+** | **-** |
| 31 | Q8IBN1_PLAF7 | Rhoptry-associated leucine zipper-like protein 1 | **+** | **-** |
| 32 | Q8IFM0_PLAF7 | Plasmodium RESA N-terminal domain-containing protein | **+** | **+** |
| 33 | ACT1_PLAF7 | Actin-1 | **-** | **+** |
| 34 | Q8I0U6_PLAF7 | Ring-infected erythrocyte surface antigen | **+** | **-** |
| ***Cellular signaling proteins*** | | | | |
| 35 | Q8IL80_PLAF7 | Thioredoxin peroxidase 1 | **+** | **+** |
| 36 | Q8IDH5_PLAF7 | Thioredoxin-related protein, putative | **+** | **-** |
| 37 | Q8IIR9_PLAF7 | Casein kinase 2, alpha subunit | **+** | **-** |
| 38 | Q7KQK6_PLAF7 | GTP-binding nuclear protein | **+** | **-** |
| 39 | Q8IB31_PLAF7 | Ubiquitin-like domain-containing protein | **+** | **-** |
| 40 | C0H552_PLAF7 | WD repeat-containing protein | **+** | **-** |
| 41 | 1433I_PLAF7 | 14-3-3 protein I | **+** | **-** |
| ***Energy metabolism and protein synthesis*** | | | | |
| 42 | Q8IDB0_PLAF7 | 40S ribosomal protein S15 | **+** | **+** |
| 43 | Q8IDI5_PLAF7 | 60S ribosomal protein L17, putative | **+** | **+** |
| 44 | Q8IDS6_PLAF7 | 60S ribosomal protein L18a | **+** | **+** |
| 45 | Q8I3T9_PLAF7 | 60S ribosomal protein L2 | **+** | **-** |
| 46 | Q8IJC6_PLAF7 | 60S ribosomal protein L3 | **+** | **+** |
| 47 | Q8IE85_PLAF7 | 60S ribosomal protein L6, putative | **+** | **+** |
| 48 | Q8ILL2_PLAF7 | 60S ribosomal protein L7a | **+** | **-** |
| 49 | Q8IJR6_PLAF7 | Autophagy-related protein 18 | **+** | **-** |
| 50 | Q8I5A0_PLAF7 | Dihydrolipoyl dehydrogenase | **+** | **-** |
| 51 | PLM1_PLAF7 | Plasmepsin I | **+** | **+** |
| 52 | PLM3_PLAF7 | Plasmepsin III | **+** | **-** |
| 53 | C6KST3_PLAF7 | Proteasome subunit alpha type-2, putative | **+** | **+** |
| 54 | Q8I0V4_PLAF7 | Endoplasmin, putative | **+** | **+** |
| 55 | Q8II60_PLAF7 | 26S protease regulatory subunit 6A, putative | **+** | **-** |
| 56 | Q8I1V1_PLAF7 | 26S protease regulatory subunit 6B, putative | **+** | **-** |
| 57 | O96153_PLAF7 | 26S proteasome regulatory subunit RPN1, putative | **+** | **-** |
| 58 | Q8IAX5_PLAF7 | 40S ribosomal protein S16, putative | **+** | **-** |
| 59 | O97248_PLAF7 | 40S ribosomal protein S23, putative | **+** | **-** |
| 60 | Q8IDR9_PLAF7 | 40S ribosomal protein S6 | **+** | **-** |
| 61 | Q8IM10_PLAF7 | 40S ribosomal protein S8 | **+** | **-** |
| 62 | Q8I3R0_PLAF7 | 40S ribosomal protein S9, putative | **+** | **-** |
| 63 | Q8I0V3_PLAF7 | 60 kDa chaperonin | **+** | **-** |
| 64 | Q8II61_PLAF7 | 60S acidic ribosomal protein P0 | **+** | **-** |
| 65 | C6KSY6_PLAF7 | 60S ribosomal protein L19 | **+** | **-** |
| 66 | Q8ILK3_PLAF7 | 60S ribosomal protein L21 | **+** | **-** |
| 67 | Q8IIB4_PLAF7 | 60S ribosomal protein L35ae, putative | **+** | **-** |
| 68 | O97250_PLAF7 | 60S ribosomal protein L7, putative | **+** | **-** |
| 69 | Q8IJ34_PLAF7 | ADP/ATP translocase | **+** | **-** |
| 70 | Q8IBD0_PLAF7 | CRA domain-containing protein, putative | **+** | **-** |
| 71 | Q8IEA6_PLAF7 | dihydrolipoyllysine-residue succinyltransferase | **+** | **-** |
| 72 | Q8IAX8_PLAF7 | DNA/RNA-binding protein Alba 1 | **+** | **-** |
| 73 | Q8IJX8_PLAF7 | DNA/RNA-binding protein Alba 3 | **+** | **-** |
| 74 | A0A5K1K8Y8_PLAF7 | DNA/RNA-binding protein Alba 4 | **+** | **-** |
| 75 | Q8I2K5_PLAF7 | Elongation factor Tu, putative | **+** | **-** |
| 76 | Q8IE22_PLAF7 | ER lumen protein retaining receptor 1, putative | **+** | **-** |
| 77 | ALF_PLAF7 | Fructose-bisphosphate aldolase | **+** | **-** |
| 78 | Q8ILT0_PLAF7 | Glutamate dehydrogenase | **+** | **-** |
| 79 | Q8IKK7_PLAF7 | Glyceraldehyde-3-phosphate dehydrogenase | **+** | **-** |
| 80 | Q8I2W3_PLAF7 | Nucleosome assembly protein | **+** | **-** |
| 81 | Q8IDR1_PLAF7 | phosphoenolpyruvate carboxykinase (ATP) | **+** | **-** |
| 82 | Q8IFP2_PLAF7 | 40S ribosomal protein S19 | **+** | **+** |
| 83 | Q8IKH8_PLAF7 | 40S ribosomal protein S3 | **+** | **+** |
| 84 | Q8IL02_PLAF7 | 40S ribosomal protein S5 | **+** | **+** |
| 85 | Q8ILV2_PLAF7 | 60S ribosomal protein L10, putative | **+** | **+** |
| 86 | Q8IE09_PLAF7 | 60S ribosomal protein L23, putative | **+** | **+** |
| 87 | Q8I431_PLAF7 | 60S ribosomal protein L4 | **+** | **+** |
| 88 | Q76NM3_PLAF7 | L-lactate dehydrogenase | **+** | **+** |
| 89 | Q8ILE8_PLAF7 | 60S ribosomal protein L14, putative | **-** | **+** |
| 90 | C0H4A6_PLAF7 | Ribosomal protein L15 | **-** | **+** |
| 91 | Q8IEQ1_PLAF7 | 26S protease regulatory subunit 10B, putative | **+** | **-** |
| ***Transport proteins*** | | | | |
| 92 | Q8I6Z4_PLAF7 | ABC transporter E family member 1, putative | **+** | **-** |
| 93 | Q8II36_PLAF7 | Aquaglyceroporin | **+** | **-** |
| 94 | Q8IIF0_PLAF7 | Exported protein 1 | **+** | **+** |
| 95 | Q8IKC8_PLAF7 | Exported protein 2 | **+** | **+** |
| 96 | Q8I3M5_PLAF7 | Karyopherin beta | **+** | **-** |
| 97 | Q8I305_PLAF7 | Major facilitator superfamily-related transporter, putative | **+** | **-** |
| 98 | Q7K6A5_PLAF7 | Multidrug resistance protein 1 | **+** | **-** |
| 99 | Q8ILA1_PLAF7 | Translocon component PTEX150 | **+** | **+** |
| 100 | Q8IKJ0_PLAF7 | V-type proton ATPase subunit | **+** | **-** |
| 101 | Q8IAQ8_PLAF7 | V-type proton ATPase subunit a, putative | **+** | **-** |
| 102 | ARF1_PLAF7 | ADP-ribosylation factor 1 | **+** | **-** |
| 103 | Q9TY94_PLAF7 | ATP-dependent RNA helicase UAP56 | **+** | **-** |
| 104 | Q8I5L6_PLAF7 | Clathrin heavy chain | **+** | **-** |
| 105 | Q8IIK1_PLAF7 | Folate transporter 2 | **+** | **-** |
| 106 | O77389_PLAF7 | Formate-nitrite transporter | **+** | **-** |
| 107 | Q7KQK6_PLAF7 | H(+)-exporting diphosphatase | **+** | **-** |
| 108 | Q7KWJ5_PLAF7 | Hexose transporter | **+** | **-** |
| 109 | Q8IBD1_PLAF7 | Sporozoite and liver stage tryptophan-rich protein, putative | **+** | **-** |
| 110 | Q8IIU7_PLAF7 | Translocon component PTEX88 | **+** | **-** |
| 111 | Q8IDB8_PLAF7 | YOP1-like protein, putative | **+** | **-** |
| 112 | Q8I0P6_PLAF7 | Elongation factor 1-alpha | **+** | **+** |
| 113 | A0A5K1K7X4_PLAF7 | Early transcribed membrane protein 5 | **-** | **+** |
| 114 | A0A143ZZK9_PLAF7 | Non-SERCA-type Ca2+ -transporting P-ATPase | **-** | **+** |

Note: The condition column indicates whether the protein was identified in non-deficient or G6PD-deficient states, or if it was present in both conditions. A (+) signifies presence, while a (-) signifies absence.

**Supplementary** **table 6.** Protein identified in conglomerate 2 of erythrocytes with the schizont forms of *P. falciparum FCB-2*

| ***Conglomerate 2 Schizont*** | | | | |
| --- | --- | --- | --- | --- |
| ***N°*** | ***ID*** | ***Protein name*** | ***Condition*** | |
|  |  |  | ***Control*** | ***d-G6PD*** |
| ***Proteins involved in DNA and RNA processing*** | | | | |
| 1 | Q8II73_PLAF7 | Spermidine synthase | **+** | **-** |
| 2 | C6KSV9_PLAF7 | SWIB/MDM2 domain-containing protein | **+** | **-** |
| 3 | A0A5K1K910_PLAF7 | aspartate carbamoyltransferase | **+** | **-** |
| 4 | Q8IIW0_PLAF7 | Chromatin remodeling protein | **+** | **-** |
| 5 | O97320_PLAF7 | Histone H2A | **+** | **-** |
| 6 | Q8IIV1_PLAF7 | Histone H2B | **+** | **+** |
| 7 | C6KSV0_PLAF7 | Histone H3 | **+** | **-** |
| 8 | Q8IIV2_PLAF7 | Histone H4 | **+** | **+** |
| 9 | Q8IE67_PLAF7 | Phosphoribosylpyrophosphate synthetase | **+** | **-** |
| 10 | Q8I2Z8_PLAF7 | Probable ATP-dependent 6-phosphofructokinase | **+** | **+** |
| 11 | Q6ZLZ9_PLAF7 | Tubulin alpha chain | **+** | **-** |
| 12 | TBB_PLAF7 | Tubulin beta chain | **+** | **-** |
| ***Adhesion proteins*** | | | | |
| 13 | O77310_PLAF7 | Cytoadherence linked asexual protein 3.1 | **+** | **+** |
| 14 | O77309_PLAF7 | Cytoadherence linked asexual protein 3.2 | **+** | **-** |
| 15 | Q8I2G2_PLAF7 | Cytoadherence linked asexual protein 9 | **+** | **-** |
| 16 | Q8I2B4_PLAF7 | Erythrocyte binding antigen-181 | **+** | **-** |
| 17 | Q8I0U8_PLAF7 | Merozoite surface protein 1 | **+** | **-** |
| 18 | MSP9_PLAF7 | Merozoite surface protein 9 | **+** | **+** |
| 19 | RH1_PLAF7 | Reticulocyte-binding protein homolog 1 | **+** | **-** |
| 20 | P113_PLAF7 | Surface protein P113 | **+** | **-** |
| 21 | PF92_PLAF7 | Merozoite surface protein P92 | **-** | **+** |
| ***Effector proteins*** | | | | |
| 22 | ENO_PLAF7 | Enolase | **+** | **+** |
| 23 | Q8IIJ8_PLAF7 | Heat shock protein 101 | **+** | **-** |
| 24 | Q8II24_PLAF7 | Heat shock protein 70 | **+** | **+** |
| 25 | Q8IC05_PLAF7 | Heat shock protein 90 | **+** | **-** |
| 26 | Q8III6_PLAF7 | Heat shock protein 90, putative | **+** | **-** |
| 27 | Q8IKS4_PLAF7 | Hemolysin III | **+** | **-** |
| 28 | C0H571_PLAF7 | High molecular weight rhoptry protein 2 | **+** | **+** |
| 29 | Q8I395_PLAF7 | High molecular weight rhoptry protein 3 | **+** | **+** |
| 30 | Q8IL88_PLAF7 | HSP40, subfamily A | **+** | **-** |
| 31 | Q8I3L4_PLAF7 | Long-chain-fatty-acid--CoA ligase | **+** | **-** |
| 32 | ACT1_PLAF7 | Actin-1 | **+** | **-** |
| 33 | Q8IEI6_PLAF7 | EMP1-trafficking protein | **+** | **+** |
| 34 | Q8IB03_PLAF7 | Chaperone protein ClpB1 | **+** | **-** |
| 35 | Q8IFM0_PLAF7 | Plasmodium RESA N-terminal domain-containing protein | **+** | **+** |
| 36 | Q8IBZ2_PLAF7 | Prodrug activation and resistance esterase | **+** | **-** |
| 37 | Q8IKV6_PLAF7 | Rhoptry neck protein 2 | **+** | **-** |
| 38 | Q8I4R5_PLAF7 | Rhoptry neck protein 3 | **+** | **+** |
| 39 | Q8ILZ1_PLAF7 | Rhoptry-associated protein 1 | **+** | **+** |
| 40 | Q8I484_PLAF7 | Rhoptry-associated protein 2 | **+** | **+** |
| 41 | Q8I485_PLAF7 | Rhoptry-associated protein 3 | **+** | **+** |
| 42 | Q8I2G1_PLAF7 | Ring-exported protein 1 | **+** | **-** |
| 43 | Q8I0U6_PLAF7 | Ring-infected erythrocyte surface antigen | **+** | **+** |
| 44 | Q8IIV8_PLAF7 | Thioredoxin-like mero protein | **+** | **-** |
| ***Cellular signaling proteins*** | | | | |
| 45 | 1433I_PLAF7 | 14-3-3 protein I | **+** | **-** |
| 46 | CDPK1_PLAF7 | Calcium-dependent protein kinase 1 | **+** | **-** |
| 47 | Q8IEN3_PLAF7 | Carbamoyl phosphate synthetase | **+** | **-** |
| 48 | Q8II87_PLAF7 | CRAL/TRIO domain-containing protein, putative | **+** | **-** |
| 49 | Q8I0V4_PLAF7 | Endoplasmin, putative | **+** | **-** |
| 50 | Q7KQK6_PLAF7 | GTP-binding nuclear protein | **+** | **-** |
| 51 | Q8IB94_PLAF7 | HECT-like E3 ubiquitin ligase, putative | **+** | **-** |
| 52 | Q7KQK2_PLAF7 | Polyubiquitin | **+** | **+** |
| 53 | Q8IKJ1_PLAF7 | Serine/threonine protein phosphatase UIS2, putative | **+** | **-** |
| 54 | THIO2_PLAF7 | Thioredoxin 2 | **+** | **-** |
| 55 | Q8IL80_PLAF7 | Thioredoxin peroxidase 1 | **+** | **+** |
| 56 | Q8IDH5_PLAF7 | Thioredoxin-related protein, putative | **+** | **-** |
| 57 | Q8IB31_PLAF7 | Ubiquitin-like domain-containing protein | **+** | **+** |
| 58 | C6KT34_PLAF7 | Cell division cycle protein 48 homologue, putative | **+** | **-** |
| ***Energy metabolism and protein synthesis*** | | | | |
| 59 | Q8IJM0_PLAF7 | 26S proteasome regulatory subunit p55, putative | **+** | **-** |
| 60 | O96153_PLAF7 | 26S proteasome regulatory subunit RPN1, putative | **+** | **+** |
| 61 | Q8ID28_PLAF7 | 26S proteasome regulatory subunit RPN11, putative | **+** | **-** |
| 62 | O77345_PLAF7 | 26S proteasome regulatory subunit RPN12, putative | **+** | **-** |
| 63 | Q8IKH3_PLAF7 | 26S proteasome regulatory subunit RPN2, putative | **+** | **-** |
| 64 | A0A5K1K9F3_PLAF7 | 26S proteasome regulatory subunit RPN3, putative | **+** | **-** |
| 65 | Q8IM66_PLAF7 | 26S proteasome regulatory subunit RPN6 | **+** | **-** |
| 66 | Q8IJA0_PLAF7 | 26S proteasome regulatory subunit RPN9, putative | **+** | **-** |
| 67 | Q8IDB0_PLAF7 | 40S ribosomal protein S15 | **+** | **-** |
| 68 | Q8IAX5_PLAF7 | 40S ribosomal protein S16, putative | **+** | **-** |
| 69 | Q8IIA2_PLAF7 | 40S ribosomal protein S18, putative | **+** | **-** |
| 70 | Q8IFP2_PLAF7 | 40S ribosomal protein S19 | **+** | **-** |
| 71 | O97248_PLAF7 | 40S ribosomal protein S23, putative | **+** | **-** |
| 72 | Q8I3R6_PLAF7 | 40S ribosomal protein S24 | **+** | **-** |
| 73 | Q8IKH8_PLAF7 | 40S ribosomal protein S3 | **+** | **+** |
| 74 | Q8IIU8_PLAF7 | 40S ribosomal protein S4 | **+** | **-** |
| 75 | Q8IL02_PLAF7 | 40S ribosomal protein S5 | **+** | **-** |
| 76 | Q8IBN5_PLAF7 | 40S ribosomal protein S5, putative | **+** | **-** |
| 77 | Q8IM10_PLAF7 | 40S ribosomal protein S8 | **+** | **-** |
| 78 | Q8I3R0_PLAF7 | 40S ribosomal protein S9, putative | **+** | **-** |
| 79 | Q8I0V3_PLAF7 | 60 kDa chaperonin | **+** | **+** |
| 80 | Q8II61_PLAF7 | 60S acidic ribosomal protein P0 | **+** | **-** |
| 81 | Q8ILV2_PLAF7 | 60S ribosomal protein L10, putative | **+** | **+** |
| 82 | Q8I3T8_PLAF7 | 60S ribosomal protein L12, putative | **+** | **-** |
| 83 | Q8IAX6_PLAF7 | 60S ribosomal protein L13 | **+** | **-** |
| 84 | Q8ILE8_PLAF7 | 60S ribosomal protein L14, putative | **+** | **-** |
| 85 | Q8IDI5_PLAF7 | 60S ribosomal protein L17, putative | **+** | **-** |
| 86 | C0H5G3_PLAF7 | 60S ribosomal protein L18-2, putative | **+** | **-** |
| 87 | Q8IDS6_PLAF7 | 60S ribosomal protein L18a | **+** | **-** |
| 88 | C6KSY6_PLAF7 | 60S ribosomal protein L19 | **+** | **-** |
| 89 | Q8I3T9_PLAF7 | 60S ribosomal protein L2 | **+** | **-** |
| 90 | Q8ILK3_PLAF7 | 60S ribosomal protein L21 | **+** | **-** |
| 91 | Q8IE09_PLAF7 | 60S ribosomal protein L23, putative | **+** | **-** |
| 92 | C6KT23_PLAF7 | 60S ribosomal protein L27a, putative | **+** | **-** |
| 93 | Q8IJC6_PLAF7 | 60S ribosomal protein L3 | **+** | **+** |
| 94 | Q8IJK8_PLAF7 | 60S ribosomal protein L30e, putative | **+** | **-** |
| 95 | Q8IIB4_PLAF7 | 60S ribosomal protein L35ae, putative | **+** | **+** |
| 96 | Q8I431_PLAF7 | 60S ribosomal protein L4 | **+** | **-** |
| 97 | Q8ILL3_PLAF7 | 60S ribosomal protein L5, putative | **+** | **-** |
| 98 | Q8IE85_PLAF7 | 60S ribosomal protein L6, putative | **+** | **-** |
| 99 | O97250_PLAF7 | 60S ribosomal protein L7, putative | **+** | **-** |
| 100 | Q8ILL2_PLAF7 | 60S ribosomal protein L7a | **+** | **-** |
| 101 | C6KTB4_PLAF7 | Acetyl-CoA synthetase, putative | **+** | **-** |
| 102 | Q8I6Z1_PLAF7 | Acyl-CoA synthetase | **+** | **-** |
| 103 | Q8IJ34_PLAF7 | ADP/ATP translocase | **+** | **-** |
| 104 | Q8IBD0_PLAF7 | CRA domain-containing protein, putative | **+** | **-** |
| 105 | Q8I5S6_PLAF7 | Eukaryotic translation initiation factor 3 subunit A | **+** | **-** |
| 106 | Q8I2X0_PLAF7 | Eukaryotic translation initiation factor 3 subunit F, putative | **+** | **-** |
| 107 | Q8I1Q5_PLAF7 | Eukaryotic translation initiation factor 3 subunit M | **+** | **-** |
| 108 | Q8I3T4_PLAF7 | FACT complex subunit | **+** | **-** |
| 109 | ALF_PLAF7 | Fructose-bisphosphate aldolase | **+** | **-** |
| 110 | Q8ILT0_PLAF7 | Glutamate dehydrogenase | **+** | **+** |
| 111 | O97245_PLAF7 | Glycerol-3-phosphate dehydrogenase | **+** | **-** |
| 112 | Q8IKF6_PLAF7 | Kelch domain-containing protein, putative | **+** | **+** |
| 113 | RL44_PLAF7 | Large ribosomal subunit protein P2 | **+** | **-** |
| 114 | AMPL_PLAF7 | Leucine aminopeptidase | **+** | **-** |
| 115 | Q76NM3_PLAF7 | L-lactate dehydrogenase | **+** | **+** |
| 116 | C6KSX2_PLAF7 | Nucleolar GTP-binding protein 1 | **+** | **-** |
| 117 | Q8ILM2_PLAF7 | Nucleolar GTP-binding protein 2 | **+** | **-** |
| 118 | Q8IJV7_PLAF7 | Nucleolar protein 5, putative | **+** | **-** |
| 119 | Q8III3_PLAF7 | Nucleolar protein 56, putative | **+** | **-** |
| 120 | PLM1_PLAF7 | Plasmepsin I | **+** | **+** |
| 121 | PLM4_PLAF7 | Plasmepsin IV | **+** | **-** |
| 122 | Q8I1X5_PLAF7 | Pre-mRNA-processing-splicing factor 8, putative | **+** | **-** |
| 123 | Q8IFP1_PLAF7 | Pre-mRNA-splicing helicase BRR2, putative | **+** | **-** |
| 124 | Q8I374_PLAF7 | Proteasome activator 28 subunit beta, putative | **+** | **-** |
| 125 | Q8IDG2_PLAF7 | Proteasome subunit alpha type | **+** | **-** |
| 126 | Q8IK90_PLAF7 | Proteasome subunit alpha type-1, putative | **+** | **-** |
| 127 | C6KST3_PLAF7 | Proteasome subunit alpha type-2, putative | **+** | **-** |
| 128 | Q8IAR3_PLAF7 | Proteasome subunit alpha type-6, putative | **+** | **-** |
| 129 | Q8IJT1_PLAF7 | Proteasome subunit beta | **+** | **-** |
| 130 | Q8IHQ8_PLAF7 | Ribosome assembly protein 4, putative | **+** | **-** |
| 131 | Q8IKF0_PLAF7 | RNA helicase | **+** | **-** |
| 132 | RS3A_PLAF7 | Small ribosomal subunit protein eS1 | **+** | **-** |
| 133 | Q8I574_PLAF7 | Splicing factor 3B subunit 3, putative | **+** | **-** |
| 134 | C0H5I7_PLAF7 | T-complex protein 1 subunit delta | **+** | **-** |
| 135 | O96220_PLAF7 | T-complex protein 1 subunit theta | **+** | **-** |
| 136 | C6KST5_PLAF7 | T-complex protein 1 subunit zeta | **+** | **-** |
| 137 | O96127_PLAF7 | Translation-enhancing factor | **+** | **-** |
| 138 | Q8IAU1_PLAF7 | ATP-dependent RNA helicase DBP1, putative | **+** | **-** |
| 139 | Q8IL13_PLAF7 | ATP-dependent RNA helicase DDX5, putative | **+** | **-** |
| 140 | Q8IAX8_PLAF7 | DNA/RNA-binding protein Alba 1 | **+** | **-** |
| 141 | Q8IJX8_PLAF7 | DNA/RNA-binding protein Alba 3 | **+** | **-** |
| 142 | Q8IKK7_PLAF7 | Glyceraldehyde-3-phosphate dehydrogenase | **+** | **-** |
| 143 | Q8I546_PLAF7 | DUF4149 domain-containing protein | **-** | **+** |
| 144 | Q8IEQ1_PLAF7 | 26S protease regulatory subunit 10B, putative | **+** | **-** |
| 145 | Q8IJW0_PLAF7 | 26S protease regulatory subunit 4, putative | **+** | **-** |
| 146 | Q8II60_PLAF7 | 26S protease regulatory subunit 6A, putative | **+** | **-** |
| 147 | Q8I1V1_PLAF7 | 26S protease regulatory subunit 6B, putative | **+** | **-** |
| 148 | Q8IEK3_PLAF7 | 26S protease regulatory subunit 7, putative | **+** | **-** |
| 149 | Q8I4U5_PLAF7 | 26S protease regulatory subunit 8, putative | **+** | **-** |
| 150 | C6KT09_PLAF7 | Malate:quinone oxidoreductase | **+** | **-** |
| ***Transport proteins*** | | | | |
| 151 | Q8I6Z4_PLAF7 | ABC transporter E family member 1, putative | **+** | **-** |
| 152 | ARF1_PLAF7 | ADP-ribosylation factor 1 | **+** | **-** |
| 153 | Q8II36_PLAF7 | Aquaglyceroporin | **+** | **+** |
| 154 | Q8I5L6_PLAF7 | Clathrin heavy chain | **+** | **-** |
| 155 | Q8IHR4_PLAF7 | Dynamin-like protein | **+** | **-** |
| 156 | Q8I5M3_PLAF7 | Dynamin-like protein, putative | **+** | **-** |
| 157 | Q9NLB8_PLAF7 | EH domain-containing protein | **+** | **-** |
| 158 | Q8IIF0_PLAF7 | Exported protein 1 | **+** | **+** |
| 159 | Q8IKC8_PLAF7 | Exported protein 2 | **+** | **+** |
| 160 | O77389_PLAF7 | Formate-nitrite transporter | **+** | **-** |
| 161 | Q8IKR1_PLAF7 | H(+)-exporting diphosphatase | **+** | **+** |
| 162 | Q7KWJ5_PLAF7 | Hexose transporter | **+** | **-** |
| 163 | Q8I3M5_PLAF7 | Karyopherin beta | **+** | **+** |
| 164 | Q8I305_PLAF7 | Major facilitator superfamily-related transporter, putative | **+** | **-** |
| 165 | Q7K6A5_PLAF7 | Multidrug resistance protein 1 | **+** | **+** |
| 166 | A0A143ZZK9_PLAF7 | Non-SERCA-type Ca2+ -transporting P-ATPase | **+** | **-** |
| 167 | Q8II72_PLAF7 | Parasitophorous vacuolar protein 1 | **+** | **-** |
| 168 | Q8I5E9_PLAF7 | Parasitophorous vacuolar protein 2 | **+** | **-** |
| 169 | CRT_PLAF7 | Putative chloroquine resistance transporter | **+** | **-** |
| 170 | 6Q76NM4_PLAF7 | Ras-related protein Rab-11A | **+** | **-** |
| 171 | Q8I5A9_PLAF7 | Ras-related protein Rab-2 | **+** | **-** |
| 172 | Q8I298_PLAF7 | StAR-related lipid transfer protein | **+** | **-** |
| 173 | Q8ILA1_PLAF7 | Translocon component PTEX150 | **+** | **-** |
| 174 | Q8IIU7_PLAF7 | Translocon component PTEX88 | **+** | **-** |
| 175 | VATA_PLAF7 | V-type proton ATPase catalytic subunit A | **+** | **-** |
| 176 | Q8IAQ8_PLAF7 | V-type proton ATPase subunit a, putative | **+** | **+** |
| 177 | Q8IDB8_PLAF7 | YOP1-like protein, putative | **+** | **-** |
| 178 | Q8IJ76_PLAF7 | Early transcribed membrane protein 10.2 | **+** | **-** |
| 179 | A0A5K1K7X4_PLAF7 | Early transcribed membrane protein 5 | **+** | **-** |
| 180 | Q8I0P6_PLAF7 | Elongation factor 1-alpha | **+** | **-** |
| 181 | Q8IKW5_PLAF7 | Elongation factor 2 | **+** | **-** |
| 182 | Q8I0V2_PLAF7 | ATP synthase subunit beta | **-** | **+** |
| 183 | Q8I487_PLAF7 | Skeleton-binding protein 1 | **+** | **-** |

Note: The condition column indicates whether the protein was identified in non-deficient or G6PD-deficient states, or if it was present in both conditions. A (+) signifies presence, while a (-) signifies absence.

***Figure S2.*** Functional classification of *Plasmodium falciparum* proteins identified in Conglomerate-1. Proteins were detected in infected erythrocytes from non-deficient controls (blue bars) and G6PD-deficient donors (green bars), across the three intraerythrocytic stages (Ring, Trophozoite, Schizont). G6PD deficiency was associated with a global reduction in protein identifications, most notably in effector proteins and metabolic functions, particularly during the trophozoite and schizont stages.

***Figure S3.*** Functional classification of *Plasmodium falciparum* proteins identified in Conglomerate-2. Bar graphs illustrate the number of proteins detected in non-deficient (blue) and G6PD-deficient (green) erythrocytes, categorized by parasite stage (Ring, Trophozoite, Schizont). In comparison to the control condition, G6PD-deficient samples consistently exhibited a reduced number of proteins, particularly in categories associated with parasite metabolism, transcriptional regulation, and host interaction mechanisms.
